# Excipient of paclitaxel induces metabolic dysregulation and unfolded protein response

**DOI:** 10.1016/j.isci.2021.103170

**Published:** 2021-09-25

**Authors:** Qian Dai, Xiaolin Liu, Tao He, Chao Yang, Jinfeng Jiang, Yin Fang, Zhoukai Fu, Yuan Yuan, Shujun Bai, Tong Qiu, Rutie Yin, Ping Ding, Jie Chen, Qintong Li

**Affiliations:** 1 Departments of Obstetrics & Gynecology and Pediatrics, West China Second University Hospital, Key Laboratory of Birth Defects and Related Diseases of Women and Children, Ministry of Education, Center of Growth, Metabolism and Aging, State Key Laboratory of Biotherapy and Collaborative Innovation Center of Biotherapy, Sichuan University, Chengdu 610041, China; 2 Department of Breast Surgery, Clinical Research Center for Breast Diseases, West China Hospital, Sichuan University, Chengdu 610041, China; 3 Divisions of Bioinformatics & Immunology, Cunde Therapeutics, Chengdu 610093, China; 4 Non-coding RNA and Drug Discovery Key Laboratory of Sichuan Province, Chengdu Medical College, Chengdu 610500, China

**Keywords:** Biological sciences, Molecular biology, Cell biology, Cancer

## Abstract

Taxane-based reagents, such as Taxol, Taxotere, and Abraxane, are popular anti-cancer drugs that can differ in their clinical efficacy. This difference is generally attributed to their active pharmaceutical ingredients. Here, we report a serendipitous discovery that Taxol induces metabolic dysregulation and unfolded protein response. Surprisingly, these effects of Taxol are entirely dependent on its excipient, Cremophor EL (CrEL). We show that CrEL promotes aerobic glycolysis and in turn results in drastic upregulation of *angiopoietin like 4* (*ANGPTL4*), a major regulator of human blood lipid profile. Notably, premedication with dexamethasone further enhances the expression of *ANGPTL4*. Consistently, we find that the amplitude and frequency of increase in triglycerides is more prominent in Taxol-treated patients with breast cancer. In addition, we find that CrEL activates the unfolded protein response pathway to trigger proinflammatory gene expression and caspase/gasdermin E-dependent pyroptosis. Finally, we discuss the implications of these results in anti-cancer therapies.

## Highlights

• Cremophor EL, the excipient of chemotherapy drug Taxol, is biologically active
• Cremophor EL promotes aerobic glycolysis in cancer and primary human immune cells
• Dexamethasone and Cremophor EL may cause dyslipidemia via ANGPTL4 upregulation
• Cremophor EL promotes the unfolded protein response

## Graphical abstract

**Figure:**
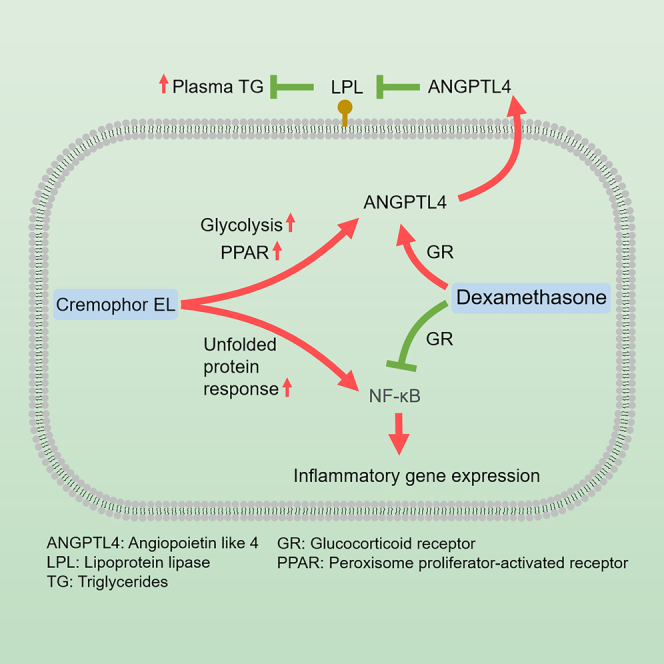

## Introduction

Paclitaxel is one of the most popular chemotherapy drugs to treat human malignancies such as lung and breast cancers. The cellular target of paclitaxel is microtubules (Kavallaris, 2010). However, the mechanism of action to explain its clinical efficacy is constantly debated in scientific community (Haschka et al., 2018). The cytotoxic effect of paclitaxel was originally attributed to its ability to stabilize microtubule polymer, resulting in cell cycle arrest at mitosis (Jordan et al., 1993). However recent studies have challenged this view and propose chromosome missegregation-embarked cell death (Zasadil et al., 2014) or the disruption of the microtubule-dependent transport of cellular cargos as the main mechanism to account for the therapeutical efficacy of paclitaxel (Poruchynsky et al., 2015).

The excipient is the key for the successful clinical translation of paclitaxel because it is practically insoluble in aqueous solution (Hennenfent and Govindan, 2006). For basic research, paclitaxel is commonly dissolved in highly polar organic solvents, such as dimethyl sulfoxide (DMSO) and ethanol, to study its function in cell culture models. However, to formulate paclitaxel as an anti-cancer drug, microemulsion and nanoemulsion are required (Ezrahi et al., 2019). In this regard, the first Food and Drug Administration (FDA)-approved, paclitaxel-based antineoplastic drug, known as Taxol, is formulated with the excipient containing the equal volume of Cremophor EL (CAS Number: 61791-12-6) and dehydrated ethanol (Adams et al., 1993). It is believed that Cremophor EL (CrEL), composed mostly of polyoxyethylene glycerol triricinoleate, forms micelles to carry paclitaxel in the systemic circulation (Ezrahi et al., 2019). In addition to paclitaxel, CrEL is also used to formulate other marketed drugs such as anesthetics propofol (Gelderblom et al., 2001). However, there are several adverse effects associated with CrEL. For example, CrEL induces hypersensitivity reactions, so severe that premedication with immunosuppressants, such as dexamethasone, is required before Taxol infusion (Gelderblom et al., 2001). Despite this countermeasure, 40% of patients still experience minor inflammatory reactions and nearly 3% experience life-threatening hypersensitivity reactions (Weiss et al., 1990; Hennenfent and Govindan, 2006; Gradishar et al., 2005). Other clinically relevant side effects, such as neurotoxicity, are also known (Mielke et al., 2006). In human plasma, CrEL is a slow clearance compound with an average half-life of 85 h (Sparreboom et al., 1998). Substantial levels of CrEL can still be detected in human plasma even at 1 week after Taxol treatment (Sparreboom et al., 1998). The plasma concentration of CrEL varies greatly among patients, ranging from 0.2‰ to 2‰ at 27 h after one single infusion of Taxol (Rischin et al., 1996; Sparreboom et al., 1999) but can be as high as 5‰ even at 72 h after infusion (Sparreboom et al., 1998). These observations demonstrate that CrEL is highly bioactive with potential long-term adverse effects. Up to date, only a few studies investigated the immune-stimulatory mechanism of CrEL (Szebeni et al., 1998; Ilinskaya et al., 2015; Weiszhar et al., 2012). Considering such a widespread use of CrEL in marketed and investigational drugs, it is somewhat surprising that the biological pathways induced by CrEL are poorly defined.

We initially aimed to disentangle various mechanisms of paclitaxel to explain its therapeutical efficacy. Unexpectedly, we found that Taxol induces aerobic glycolysis in cultured cells, a previously unrecognized phenomenon. To our surprise, we found that this effect is entirely dependent on CrEL but not paclitaxel. This observation promoted us to systematically examine the biological effects of CrEL as well as the underlying molecular mechanisms.

## Results

### Cremophor EL enhances aerobic glycolysis

**Figure 1:**
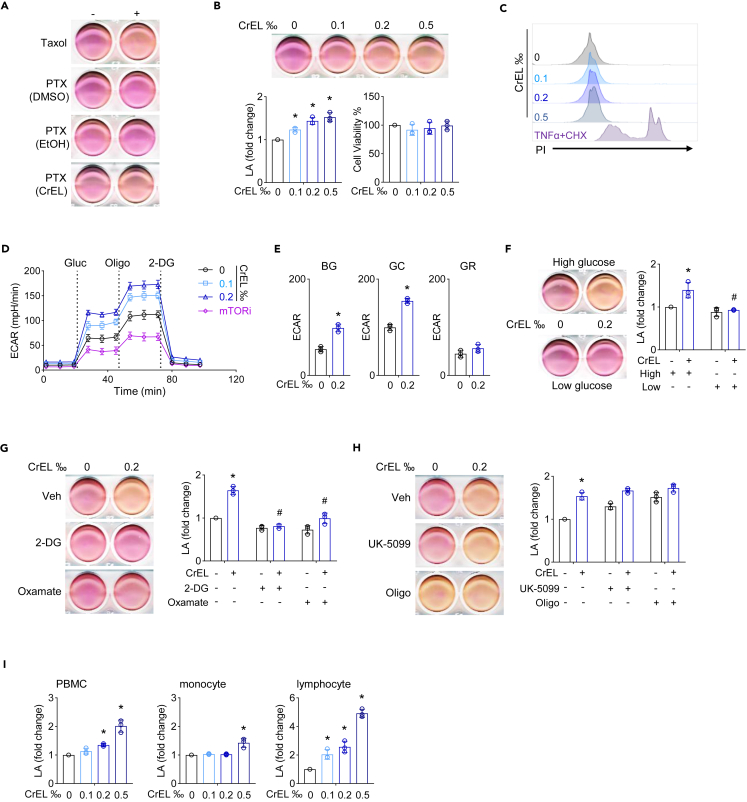
Cremophor EL enhances aerobic glycolysis (A) Extracellular acidification effect in Caov-3 cells caused by Taxol or paclitaxel (PTX) dissolved in dimethyl sulfoxide (DMSO), ethanol (EtOH), or Cremophor EL (CrEL), respectively. (B) Effect of CrEL on extracellular acidification and cellular viability. Caov-3 cells were treated with CrEL of indicated concentration overnight, and extracellular lactate concentration (LA) as well as cellular viability was measured. Data are represented as mean ± S.D. (n = 3). (C) Effect of CrEL on cellular permeability. Caov-3 cells were treated with CrEL of indicated concentration overnight and stained with propidium iodide (PI) for FACS analysis. Treatment with TNFα plus cycloheximide (CHX) was used to induce apoptosis as control of increased cellular permeability. (D) Effect of CrEL on glycolytic rate. Caov-3 cells were treated with 0.1‰, 0.2‰ of CrEL, or mTOR inhibitor (mTORi) and then subjected to Seahorse Extracellular Flux Analyzer to measure real-time extracellular acidification rate (ECAR). Glucose (Gluc), oligomycin (Oligo), and 2-DG were injected at the indicated time points. Data are represented as mean ± S.D. (n = 3). (E) Quantification of basal glycolysis (BG, following glucose injection), maximal glycolytic capacity (GC, following oligomycin injection), and glycolytic reserve (GR) in (D). Data are represented as mean ± S.D. (n = 3). (F) Effect of glucose concentration on CrEL-induced ECAR. Data are represented as mean ± S.D. (n = 3). (G) Effect of small-molecule inhibitors of glycolysis on CrEL-induced ECAR. 2-DG and oxamate inhibit the first and the last step of glycolysis, respectively. DMSO was used as treatment vehicle (Veh). Data are represented as mean ± S.D. (n = 3). (H) Effect of small-molecule inhibitors of pyruvate mitochondrial metabolism on CrEL-induced ECAR. UK-5099 inhibits the transport of pyruvate into mitochondria, and oligomycin (Oligo) inhibits ATP synthase (complex V) of the mitochondrial electron transport chain. Data are represented as mean ± S.D. (n = 3). (I) Effect of CrEL on human primary cells including peripheral blood mononuclear cells (PMBC), monocytes, and lymphocytes. ∗ denotes p < 0.05 compared to mock treatment. # denotes p < 0.05 compared to CrEL treatment (one-way ANOVA). Data are represented as mean ± S.D. (n = 3).

We set out to disentangle different mechanisms of paclitaxel. Unexpectedly, we found that Taxol treatment increased the rate of extracellular acidification in human ovarian cancer cell Caov-3 (Figure 1A). To confirm this phenomenon, we purchased analytical grade paclitaxel powder from another vendor and dissolved it with DMSO (paclitaxel/DMSO). Surprisingly, paclitaxel/DMSO had no effect on the rate of extracellular acidification (Figure 1A). Because Taxol is formulated with the excipient containing the equal volume of Cremophor EL (CAS Number: 61791-12-6, CrEL) and ethanol, we dissolved paclitaxel in ethanol (paclitaxel/ethanol). Similar to paclitaxel/DMSO, paclitaxel/ethanol had no effect on the rate of extracellular acidification (Figure 1A). In sharp contrast, paclitaxel dissolved in CrEL showed a marked increase in the rate of extracellular acidification to a similar degree as Taxol (Figure 1A). The concentration of CrEL ranges from 0.2‰ to 2‰ in patient plasma even at 24 h after Taxol infusion (Rischin et al., 1996; Sparreboom et al., 1998, 1999). We found that 0.1‰ of CrEL was sufficient to increase the rate of extracellular acidification (Figure 1B). Thus, Taxol-induced extracellular acidification is entirely dependent on CrEL.

Lactate generated by glycolysis is the main contributor of extracellular acidification. We found that up to 0.5‰ of CrEL had little effect on cellular viability (Figure 1B) or permeability (Figure 1C), indicating enhanced glycolysis instead of increased cellular proliferation or membrane leakage as the explanation for CrEL-induced extracellular acidification. Seahorse Extracellular Flux Analyzer provides a real-time measurement of cellular glycolysis by determining extracellular acidification rate (ECAR) (Divakaruni et al., 2014). Caov-3 cells were individually treated with 0.1‰, 0.2‰ of CrEL, or mTOR inhibitor (Figure 1D). Untreated cells had a similar rate of non-glycolytic acidification as cells treated with CrEL or mTOR inhibitor prior to glucose addition. After glucose was added, cells treated with CrEL showed a higher ECAR than untreated cells. As expected, cells treated with mTOR inhibitor had the lowest ECAR because mTOR signaling pathway is known to promote glycolysis. The addition of mitochondrial respiration inhibitor oligomycin revealed a higher level of maximal glycolytic capacity in cells treated with CrEL. The addition of glycolysis inhibitor 2-deoxy-D-glucose (2-DG) reduced ECAR to the same basal level. Thus, CrEL enhanced both the basal glycolytic rate (BG) and the maximal glycolytic capacity (GC) in Caov-3 cells (Figure 1E). Consistently, low-glucose medium completely blocked CrEL-induced ECAR in Caov-3 and THP-1 cells (Figures 1F and S1A). Similarly, CrEL treatment enhanced ECAR in human lung cancer cell A549, colon cancer cell HCT116, and monocytic leukemia cell THP-1 (Figure S1B). Furthermore, treatment with 2-DG (inhibitor of the first step of glycolysis) or oxamate (inhibitor of the last step of glycolysis by targeting lactate dehydrogenase) blunted CrEL-induced ECAR in THP-1 cell (Figure 1G), confirming that CrEL indeed enhances glycolysis. As expected, treatment with UK-5099 (inhibitor of pyruvate transport into mitochondria) or oligomycin (inhibitor of mitochondrial respiration) increased ECAR, but they failed to further enhance ECAR in the presence of CrEL (Figure 1H). Interestingly, oligomycin treatment increased ECAR to the same degree as CrEL (Figure 1H), demonstrating CrEL as a strong inducer of glycolysis. This also raised the possibility that CrEL might inhibit the function of mitochondria. However, this was not the case because in Seahorse Cell Mito Stress Test, cells treated with CrEL exhibited even higher basal respiration, maximal respiration, and spare respiratory capacities than untreated cells (Figure S1C). Thus, CrEL is a strong inducer of aerobic glycolysis in cancer cells.

Because Taxol is administered via intravenous infusion, immune cells in periphery blood are in intimate contact with CrEL. We thus examined the effect of CrEL on human primary immune cells. Similar to cancer cells, ECAR was increased in peripheral blood mononuclear cells (PBMCs) by CrEL treatment (Figure 1I). PBMCs are made up with monocytes (positive for the cell surface marker CD14, about 25%) and the rest mostly lymphocytes (CD14-negative). Using anti-CD14 antibody-coated magnetic beads, we obtained monocytes with 97% purity (Figure S1D). CrEL was able to induce ECAR in both monocytes and lymphocytes, but the fold of induction appeared to be higher in lymphocytes (Figure 1I). This was because lymphocytes had much lower basal ECAR than monocytes (Figure S1E). Thus, CrEL is a genuine enhancer of aerobic glycolysis in a variety of human cancer as well as primary immune cells.

### Cremophor EL activates mTOR signaling pathway to enhance aerobic glycolysis

**Figure 2:**
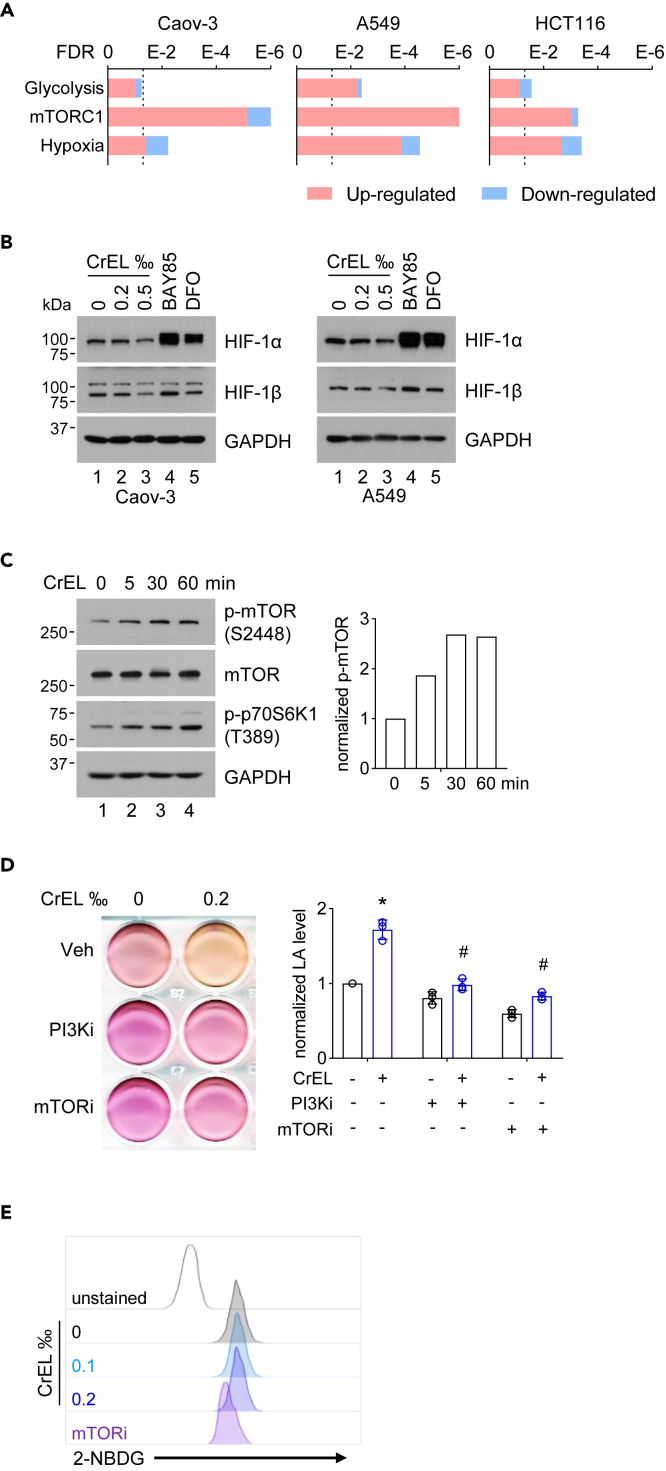
Cremophor EL activates mTOR signaling pathway to enhance aerobic glycolysis (A) Gene set enrichment analysis of differentially expressed genes induced by CrEL in indicated cancer cell lines. Vertical dashed line denotes false discovery rate (FDR) of 0.05. (B) Protein blot analysis of indicated proteins involved in hypoxia signaling. BAY 85-3934 (BAY 85) and desferrioxamine (DFO) are small-molecule inhibitors of prolyl hydroxylases to stabilize hypoxia-inducible factor (HIF). (C) Protein blot analysis of indicated proteins involved in mTOR signaling. (D) Effect of small-molecule inhibitors of mTOR signaling on CrEL-induced glycolysis. LY294002 was used as inhibitor for PI3K (PI3Ki), and INK-128 for mTOR (mTORi). Data are represented as mean ± S.D. (n = 3). (E) Effect of CrEL on glucose transport. 2-NBDG is a fluorescent glucose analog. INK-128 (mTORi) was used as a control for decreased glucose transport. ∗ denotes p < 0.05 compared to mock treatment. # denotes p < 0.05 compared to CrEL treatment (one-way ANOVA).

To understand the mechanism how CrEL promoted glycolysis, we carried out transcriptome analysis by RNA sequencing (RNA-seq) of Caov-3, A549, and HCT116 cells. Gene set enrichment analysis (GSEA) was performed using the hallmark gene set collection in the Molecular Signatures Database (Liberzon et al., 2015). Fragments per kilobase of exon model per million reads mapped (FPKM >1) and false discovery rate (FDR <0.05) were set as cutoff. For all three cell types, treatment with 0.2‰ CrEL upregulated hallmark gene sets including “glycolysis”, “mTORC1 signaling”, and “HIF1A targets” (Figures 2A and S1F). Because mammalian target of rapamycin complex 1 (mTORC1) signaling and hypoxia-inducible factor 1 (HIF-1) are known to promote glycolysis (Mossmann et al., 2018), we further examined their relevance in CrEL-induced glycolysis. HIF-1 is a heterodimer transcription factor composed of an unstable alpha subunit HIF1A and a constitutively expressed beta subunit ARNT (also known as HIF1B). At normal oxygen levels, HIF1A protein is targeted for degradation by prolyl hydroxylation, resulting in minimal HIF-1 transcriptional activity (Semenza, 2012). As expected, prolyl hydroxylase inhibitor desferrioxamine or molidustat (BAY 85-3934) stabilized HIF1A protein in different cell lines. However, 0.2‰ CrEL had little effect on HIF1A, and 0.5‰ CrEL even moderately downregulated HIF1A (Figures 2B and S1G). Thus, CrEL-induced glycolysis is unlikely dependent on HIF-1. Upon activation, mTORC1 contains mTOR phosphorylated predominantly on S2448 (Copp et al., 2009). Consistent with the upregulation of “mTORC1 signaling” by GSEA, we found that CrEL increased S2448 phosphorylation of mTOR and T389 phosphorylation of p70S6K1 (Figure 2C), a substrate of mTORC1 (Fingar and Inoki, 2012). Both CrEL and Taxol, but not paclitaxel/DMSO, were able to promote mTOR activity (Figure S1H). In addition, small-molecule inhibitor of mTOR or its upstream activator PI3K blunted CrEL-induced ECAR in THP1 and Caov-3 cells (Figures 2D and S1I). Glycolysis can be stimulated by enhanced glucose uptake. We found that the uptake rate of fluorescent glucose analog 2-NBDG was similar in untreated and CrEL-treated cells (Figures 2E and S1J), ruling out the possibility that CrEL stimulates glucose uptake. Thus, CrEL stimulates mTORC1 signaling pathway to enhance aerobic glycolysis.

### Cremophor EL and dexamethasone synergistically promote the expression of *ANGPTL4*, a major regulator of human lipid metabolism

**Figure 3:**
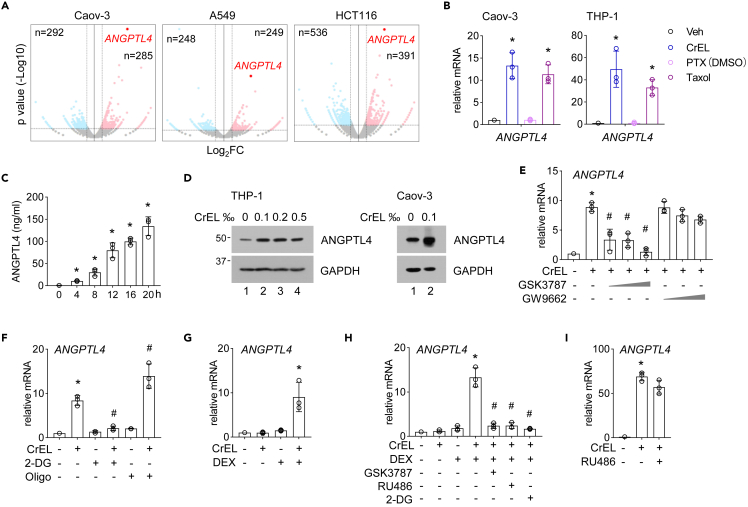
Cremophor EL and dexamethasone synergistically promote the expression of *ANGPTL4* (A) A volcano plot comparing differentially expressed gene induced by CrEL in indicated cell lines. (B) Quantitative PCR (qPCR) analysis of *ANGPTL4* expression in Caov-3 and THP-1 cells treated with DMSO (Veh), CrEL, and paclitaxel dissolved in DMSO (PTX/DMSO) or Taxol. Data are represented as mean ± S.D. (n = 3). (C) Time course analysis of secreted ANGPTL4 protein levels by enzyme-linked immunosorbent assay (ELISA). Data are represented as mean ± S.D. (n = 3). (D) Protein blot analysis of intercellular ANGPTL4 protein level. (E) Effect of small-molecule inhibitors of peroxisome proliferator-activated receptors (PPAR) on CrEL-induced *ANGPTL4* expression (qPCR). GSK3787 inhibits PPARdelta, whereas GW9662 preferentially inhibits PPARalpha/gamma. Data are represented as mean ± S.D. (n = 3). (F) Effect of small-molecule inhibitors of glycolysis on CrEL-induced *ANGPTL4* expression (qPCR). Data are represented as mean ± S.D. (n = 3). (G) Effect of dexamethasone (DEX) on CrEL-induced *ANGPTL4* expression (qPCR). Data are represented as mean ± S.D. (n = 3). (H) Effect of small-molecule inhibitors of PPARdelta (GSK3787), GR (RU486), or glycolysis (2-DG) on the synergistic induction of *ANGPTL4* expression by CrEL and DEX (qPCR). Data are represented as mean ± S.D. (n = 3). (I) Effect of small-molecule inhibitor of GR (RU486) on CrEL-induced *ANGPTL4* expression (qPCR). Data are represented as mean ± S.D. (n = 3). ∗ denotes p < 0.05 compared to mock treatment. # denotes p < 0.05 compared to CrEL treatment (one-way ANOVA).

Intriguingly, transcriptome analysis by RNA-seq identified *angiopoietin like 4* (*ANGPTL4*) as the most upregulated gene by CrEL in Caov-3, A549, and HCT116 cells (Figure 3A). ANGPTL4 protein functions as an endogenous inhibitor of lipoprotein lipase (LPL). LPL hydrolyzes fatty acids from triglyceride (TG)-rich lipoproteins in the bloodstream, thus serves as a major regulator of lipid metabolism by distributing TGs from the circulation to various organs. Recent large-scale human genetic studies and animal models have collectively established the central role of ANGPTL4 in the regulation of TG levels and cardiovascular diseases (Aryal et al., 2019; Dewey et al., 2016; Myocardial Infarction et al., 2016; Gusarova et al., 2018).

We investigated the mechanism how CrEL upregulates *ANGPTL4* expression. Quantitative PCR (qPCR) analysis showed that Taxol or CrEL, but not paclitaxel/DMSO, increased *ANGPTL4* expression in Caov-3 and THP-1 cells (Figure 3B). Thus, like enhanced aerobic glycolysis (Figure 1), CrEL is solely responsible for the upregulation of *ANGPTL4* expression. ANGPTL4 protein can be secreted and function both in an autocrine and paracrine manner. The enzyme-linked immunosorbent assay is used in many studies to determine extracellular level of ANGPTL4 protein (Aryal et al., 2019). Time point experiment showed that extracellular ANGPTL4 could be detected as early as 4 h after CrEL treatment (Figure 3C). Intracellular ANGPTL4 protein level was also increased (Figure 3D). ANGPTL4 was originally identified as the target gene of peroxisome proliferator-activated receptor (PPAR) alpha and gamma (Yoon et al., 2000; Kersten et al., 2000). Interestingly, CrEL-induced ANGPTL4 expression was blocked by the small-molecular inhibitor of PPARdelta (GSK3787) but not that of PPARalpha/gamma (GW9662) (Figure 3E). Because fatty acids are ligands for PPAR and glycolysis promotes lipogenesis, we asked whether upregulation of ANGPTL4 was dependent on CrEL-induced glycolysis. Treatment with glycolysis inhibitor 2-DG completely blocked CrEL-induced ANGPTL4 expression, whereas mitochondrial respiration inhibitor oligomycin further enhanced CrEL-induced ANGPTL4 expression (Figure 3F). These results rule out CrEL as a direct PPAR ligand but also uncover a link between glucose and lipid metabolism hinged upon ANGPTL4.

Anti-inflammatory drug dexamethasone is commonly used in premedication to manage CrEL-induced hypersensitivity in patients. Dexamethasone is an agonist of glucocorticoid receptor (GR). The powerful anti-inflammatory effect of dexamethasone is attributed to its ability to induce GR nuclear translocation to inhibit proinflammatory transcriptional programs such as those mediated by nuclear factor kappa B (NF-κB) (Cain and Cidlowski, 2017). Of note, the concentration of dexamethasone required to activate GR target genes is typically an order of magnitude higher than that required to transrepress proinflammatory gene expression (Mcmaster and Ray, 2008). Our transcriptome analysis revealed that CrEL induced expression levels of many proinflammatory genes such as IL6 and IL8 (Figure 5). As expected, pretreatment dexamethasone effectively blocked CrEL-induced expression of these genes (Figure 5). Importantly, at this concentration, dexamethasone failed to induce the expression of classic GR target genes GILZ and DUSP1 (Cain and Cidlowski, 2017). CrEL started to increase ANGPTL4 expression after 4 h (Figure 3C) but had minimal effect at 2 h (Figure 3G). We found that dexamethasone alone did not affect ANGPTL4 expression (Figure 3G). Interestingly, at 2 h, dexamethasone and CrEL collaboratively upregulated ANGPTL4 expression (Figure 3G) to a comparable level as CrEL alone at 8 h (Figure 3C). This synergistic induction of ANGPTL4 could also be blocked by PPARdelta antagonist (GSK3787) or GR antagonist (RU486) to a similar degree as glycolysis inhibitor 2-DG (Figure 3H). In contrast, RU486 failed to block CrEL-induced ANGPTL4 expression in the absence of dexamethasone (Figure 3I). Taken together, these results demonstrate that GR can potentiate but is not necessary for CrEL-induced ANGPTL4 expression.

### Taxol elevates the level of blood triglycerides in patients with breast cancer

To ask whether CrEL-induced ANGPTL4 is clinically relevant, we examined the lipid profiles of patients with breast cancer. Three taxane-based reagents are widely used as first-line treatment for patients with breast cancer, that is, paclitaxel formulated with either CrEL (Taxol) or solvent-free, albumin nanoparticles (Abraxane) and docetaxel formulated with polysorbate (Taxotere). In both animal models and human genetic studies, increased ANGPTL4 level is positively associated with higher level of blood TGs as well as lower high-density lipoprotein cholesterol (HDL-C) and vice versa (Aryal et al., 2019; Gusarova et al., 2018; Myocardial Infarction et al., 2016; Dewey et al., 2016). Considering that substantial levels of CrEL can still be detected in human plasma even at 1 week after Taxol treatment (Sparreboom et al., 1998), we hypothesized that dyslipidemia might occur in Taxol-treated patients with breast cancer.

**Figure 4:**
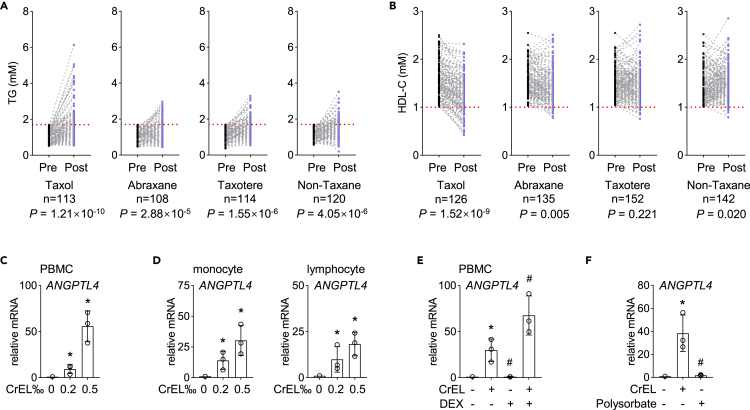
Taxol elevates the level of blood triglycerides in patients with breast cancer (A) Analysis of blood triglyceride (TG) levels in patients with breast cancer treated with taxane-based chemotherapy, including Taxol, Abraxane and Taxotere, or non-taxane-based chemotherapy (fluorouracil and epirubicin). All patients had normal levels of TG prior to chemotherapy (Pre). Blood was also drawn one week after the last round of chemotherapy (Post). Dashed line denotes 1.7 mM, the upper limit of the normal range of TG in Chinese population (Joint committee for Joint Committee for Guideline, 2018). (B) Analysis of blood high-density lipoprotein cholesterol (HDL-C) levels in patients as in (A). All patients had normal levels of HDL-C prior to chemotherapy (Pre). Dashed line denotes 1 mM, the lower limit of the normal range of HDL-C in Chinese population (Joint Committee for Guideline, 2018). (C) Effect of CrEL on *ANGPTL4* expression in human PBMCs (qPCR). Data are represented as mean ± S.D. (n = 3). (D) Effect of CrEL on *ANGPTL4* expression in human primary monocytes and lymphocytes (qPCR). Data are represented as mean ± S.D. (n = 3). (E) Effect of DEX on CrEL-induced *ANGPTL4* expression in human PBMCs (qPCR). Data are represented as mean ± S.D. (n = 3). (F) Effect of polysorbate, the excipient component of Taxotere, on *ANGPTL4* expression in THP-1 cells (qPCR). Data are represented as mean ± S.D. (n = 3). ∗ denotes p < 0.05 compared to mock treatment. # denotes p < 0.05 compared to CrEL treatment (one-way ANOVA).

Retrospective analyses of the blood lipid profile were carried out for patients with breast cancer treated with Taxol (n = 143), Abraxane (n = 144), Taxotere (n = 160), or non-taxane-based chemotherapy (n = 163). Of note, these patients were treated with otherwise identical regimens except for taxane-based drug, that is, Taxol, Abraxane or Taxotere. For all patients, blood samples were collected before chemotherapy and 1 week after the last round of chemotherapy. For each treatment regimen, approximately 75% had normal levels of TGs before chemotherapy (79% for Taxol, 75% for Abraxane, 71% for Taxotere, and 74% for non-taxane-based chemotherapy) and similar median levels of TG. First, we analyzed patient cohort with normal levels of TGs (1.7 mM) (Joint Committee for Guideline, 2018) prior to chemotherapy, containing patients treated with Taxol (n = 113), Abraxane (n = 108), Taxotere (n = 114), or non-taxane-based chemotherapy (n = 120). After chemotherapy, the increase in TG was statistically significant for all four chemotherapy regimens (p < 0.001, Figure 4A). However, the amplitude as well as the frequency of TG increase was most prominent in Taxol-treated patients. Among Taxol-treated patients, 8% (9/113) exhibited a final TG level beyond 3.4 mM, 2-fold higher than the upper limit of the normal range (1.7 mM), whereas no patients in other treatment groups exhibited such a significant increase. Then, 44% of Taxol-treated patients (50/113) also exhibited 70% increase in TG after chemotherapy, whereas only 19%, 20%, and 17% for patients treated with Abraxane, Taxotere, and non-taxane, respectively. Concomitantly, HDL-C levels were significantly lower in Taxol-treated patients (p < 0.001, Figure 4B) but not statistically significant in other treatment regimens (p > 0.001, Figure 4B). Changes in total cholesterol and low-density lipoprotein cholesterol were not statistically significant in Taxol-treated patients (p > 0.001, Figure S2A). Secondly, we analyzed patient cohort with abnormal levels of TG prior to chemotherapy (>1.7 mM), containing patients treated with Taxol (n = 30), Abraxane (n = 36), Taxotere (n = 46), or non-taxane-based chemotherapy (n = 43). Similar trends were observed as in patients with normal levels of TG prior to chemotherapy, although statistical significance was not reached due to a limited number of patients (Figure S2B). Similar to cancer cells (Figure 3), CrEL alone induced ANGPTL4 expression in PBMCs, monocytes, and lymphocytes from healthy human donors (Figures 4C and 4D), and pretreatment with dexamethasone potentiated this effect (Figure 4E). Interestingly, the excipient of Taxotere, polysorbate, failed to induce ANGPTL4 expression (Figure 4F). Thus, Taxol-treated patients exhibit dyslipidemia with much higher frequency and amplitude than other taxane-based regimens.

### Cremophor EL induces unfolded protein response to promote inflammatory reactions

**Figure 5:**
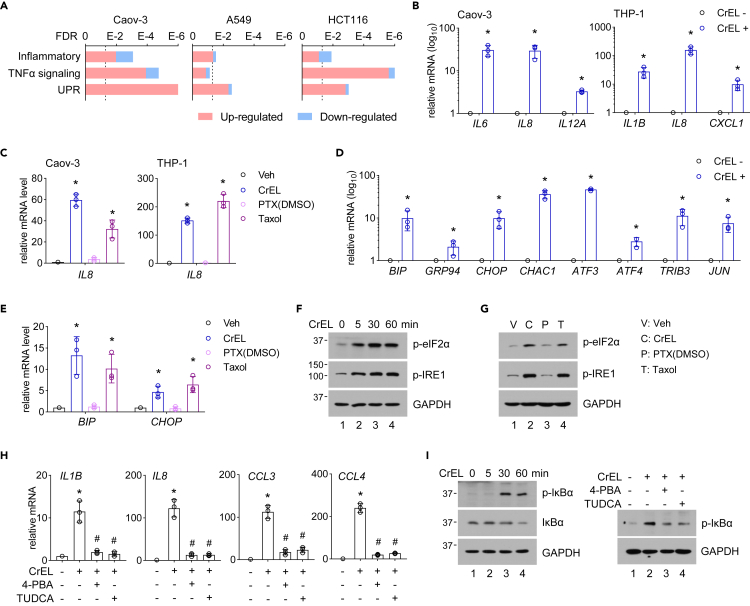
Cremophor EL induces unfolded protein response to promote inflammatory gene expression (A) Gene set enrichment analysis of differentially expressed genes induced by CrEL in indicated cancer cell lines. Vertical dashed line denotes false discovery rate (FDR) of 0.05. (B) qPCR analysis of the expression of proinflammatory genes with or without CrEL treatment in Caov-3 and THP-1 cells. Data are represented as mean ± S.D. (n = 3). (C) qPCR analysis of the expression of proinflammatory genes in Cavo-3 and THP-1 cells treated with DMSO (Veh), CrEL, and paclitaxel dissolved in DMSO (PTX/DMSO) or Taxol. Data are represented as mean ± S.D. (n = 3). (D) qPCR analysis of the expression of genes involved in unfolded protein response pathway (UPR) in THP-1 cells with or without CrEL treatment. Data are represented as mean ± S.D. (n = 3). (E) qPCR analysis of the expression of UPR genes in THP-1 cells treated with DMSO (Veh), CrEL, and paclitaxel dissolved in DMSO (PTX/DMSO) or Taxol. Data are represented as mean ± S.D. (n = 3). (F) Protein blot analysis of senor and effector proteins of UPR in THP-1 cells with or without CrEL treatment. (G) Protein blot analysis of senor and effector proteins of UPR in THP-1 cells treated with DMSO (Veh), CrEL, and paclitaxel dissolved in DMSO (PTX/DMSO) or Taxol. (H) Effect of chemical chaperons on CrEL-induced proinflammatory gene expression. Tauroursodeoxycholic acid (TUDCA) and 4-phenylbutyrate (4-PBA) are chemical chaperons to dampen UPR. Data are represented as mean ± S.D. (n = 3). (I) Effect of chemical chaperons on CrEL-induced NF-κB pathway activation. ∗ denotes p < 0.05 compared to mock treatment. # denotes p < 0.05 compared to CrEL treatment (one-way ANOVA).

Despite the clinical practice of premedication using powerful immunosuppressants such as dexamethasone, 40% of Taxol-treated patients still experience minor inflammatory reactions and nearly 3% experience life-threatening hypersensitivity reactions (Hennenfent and Govindan, 2006). These reactions are thought to be caused by CrEL, but the biological basis is poorly understood. Significantly, we found that many hallmark gene sets pertinent to inflammation were upregulated by CrEL, such as “inflammatory response” and “TNF alpha signaling via NF-κB” (Figures 5A and S3A). We confirmed that CrEL upregulated the expression of proinflammatory genes by qPCR (Figures 5B and S3B). Taxol, but not paclitaxel/DMSO, similarly induced proinflammatory gene expression (Figure 5C). As expected, treatment with dexamethasone blocked CrEL-induced proinflammatory gene expression (Figure S3C).

Interestingly, “unfold protein response” (UPR) is among the top upregulated hallmark gene sets (Figure 5A). Because UPR is known to intersect with inflammatory pathways (Hotamisligil, 2010), we investigated whether CrEL-induced hypersensitivity depends on UPR. We found that CrEL and Taxol, but not paclitaxel/DMSO, induced the expression of UPR-regulated downstream genes (Figures 5D, 5E, and S3D). CrEL or Taxol, but not paclitaxel/DMSO, also induced the activating phosphorylation of UPR sensor IRE1 and that of eIF2α, a downstream effector of UPR sensor PERK in THP-1 and Caov-3 cells (Figures 5F, 5G, and S3E). Treatment with chemical chaperones, tauroursodeoxycholic acid or 4-phenylbutyrate, to resolve UPR largely inhibited CrEL-induced proinflammatory gene expression (Figures 5H and S3F). All three canonical branches of UPR are known to suppress NF-κB inhibitors (IκBs) such as IκBα (Hotamisligil, 2010), resulting in the nuclear translocation of NF-κB to stimulate proinflammatory transcriptional program (Karin, 1999). Consistently, we found that S32 phosphorylation of IκBα was quickly stimulated within 30 min of CrEL treatment, concomitant with its proteasomal degradation (Figure 5I), and could be blocked by treatment of chemical chaperones (Figure 5I). Thus, CrEL induces unfolded protein responses to promote proinflammatory gene expression.

### Cremophor EL triggers pyroptosis, a lytic form of proinflammatory cell death

**Figure 6:**
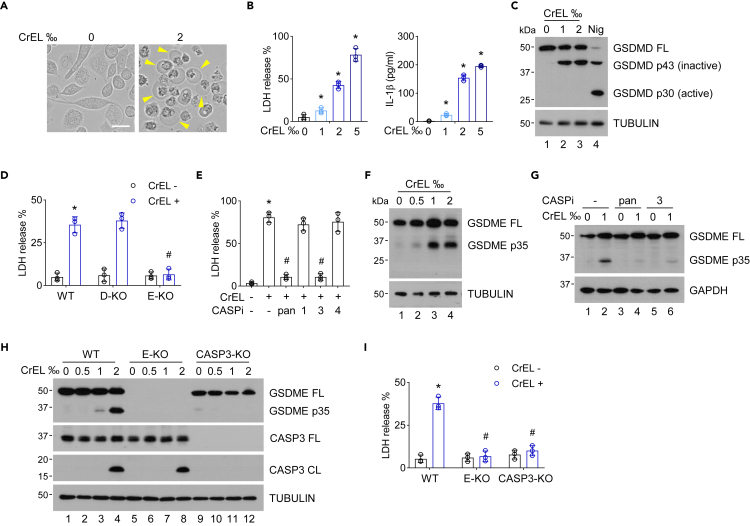
Cremophor EL triggers pyroptosis (A) Microscopic inspection of THP-1 cells with or without CrEL treatment. Arrow heads denote ballooning cell membranes, the defining morphological characteristics of pyroptosis. Scale bar, 30 μm. (B) Extracellular release of LDH and IL-1β, characteristics of pyroptosis. Data are represented as mean ± S.D. (n = 3). (C) Protein blot analysis of gasdermin D (GSDMD) cleavage. Nigericin (Nig) is the inducer of canonical, gasdermin D-mediated pyroptosis. FL, full-length. (D) Effect of gasdermin D knockout (D-KO) or gasdermin E knockout (E-KO) on CrEL-induced pyroptosis. Data are represented as mean ± S.D. (n = 3). (E) Effect of small-molecule inhibitors of caspases on CrEL-induced pyroptosis. 1, 3, and 4 denote inhibitors for pan-caspase, caspase-1, caspase-3, and caspase-4, respectively. Data are represented as mean ± S.D. (n = 3). (F) Protein blot analysis of gasdermin E (GSDME) cleavage. FL, full-length. (G) Effect of small-molecule inhibitors of caspases on CrEL-induced gasdermin E (GSDME) cleavage. (H) Effect of CrEL on gasdermin E (GSDME) and caspase-3 (CASP3) in wild-type (WT), gasdermin E knockout (E-KO), and caspase-3 knockout (CASP3-KO) cell lines. FL, full-length. CL, cleaved. (I) Effect of CrEL on pyroptosis in cells in (H). Data are represented as mean ± S.D. (n = 3). ∗ denotes p < 0.05 compared to mock treatment. # denotes p < 0.05 compared to CrEL treatment (one-way ANOVA).

The plasma concentration of CrEL can reach above 2‰ for more than 24 h in patient plasma (Sparreboom et al., 1998, 1999; Rischin et al., 1996). We hypothesized that a large amount of CrEL might induce severe UPR to trigger cell death (Hetz et al., 2020). Interestingly, microscopic examination of 2‰ CrEL-treated cells revealed extensive ballooning of the cell membrane, a defining characteristic morphology of pyroptotic cells (Figure 6A). Concomitantly, release of lactate dehydrogenase (LDH) and IL-1β into the extracellular space (hallmarks of pyroptosis) was also increased (Figure 6B). Pyroptosis is a lytic proinflammatory type of cell death, triggered by pore-forming gasdermin proteins to induce membrane permeability (Broz et al., 2020). We found that upon CrEL treatment, gasdermin D protein was cleaved into inactive p43 and p20 fragments, rather than the pyroptosis-inducing p30 fragment (Figure 6C), indicating that gasdermin D is not responsible for CrEL-induced pyroptosis. Knockout of gasdermin D by CRISPR/Cas9 genomic editing technology also failed to block increased membrane permeability by CrEL, indicated by the release of LDH (Figure 6D). The active p30 fragment of gasdermin D can be generated by caspase-1/4/5 cleavage (Shi et al., 2015; Kayagaki et al., 2015). Similarly, inhibitors of caspase-1 or 4 had little effect on CrEL-induced membrane permeability (Figure 6E). Furthermore, small-molecule inhibitors of caspase-1 or NLRP3 inflammasome fully blocked classic, nigericin-induced gasdermin D-dependent pyroptosis but failed to block CrEL-induced pyroptosis (Figure S4A). Thus, gasdermin D is not responsible for CrEL-induced pyroptosis. To rule out necroptosis, another form of inflammatory lytic form of cell death, we treated cell with inhibitors of RIPK1 or RIPK3. These inhibitors blocked TSZ-induced necroptosis but failed to block CrEL-induced cell death (Figure S4B). On the other hand, knockout of gasdermin E by CRISPR/Cas9 largely blocked CrEL-induced membrane permeability (Figure 6D). Consistently, CrEL induced the cleavage of gasdermin E into its active, pore-forming N-terminal fragment, p35 (Figure 6F). Recent studies have shown that caspase-3 cleaves gasdermin E to generate pore-forming fragment but processes gasdermin D into inactive p43 and p20 fragments (Wang et al., 2017; Rogers et al., 2017). Consistently, we found that inhibitors of caspase-3 or pan-caspase eliminated CrEL-induced pyroptosis (Figure 6E), as well as cleavage of gasdermin E protein (Figure 6G). Knockout of caspase-3 by CRISPR/Cas9 genomic editing technology also blocked cleavage of gasdermin E as well as LDH release (Figures 6H and 6I). Similarly, CrEL induced characteristic morphology of pyroptosis in gasdermin E-expressing HeLa but not in RKO cells lack of endogenous expression of gasdermin E (Figures S4C and S4D). Of note, primary human monocytes also express significant amount of gasdermin E (Figure S4E). Thus, clinically relevant concentration of CrEL at its high range can trigger a lytic form of proinflammatory cell death, pyroptosis.

## Discussion

The side effects of chemotherapy drugs are, in general, poorly understood. Here, we report a serendipitous finding that Taxol, a popular anticancer drug, stimulates aerobic glycolysis and hyperlipidemia. Surprisingly, we find that these metabolic effects are entirely dependent on the excipient component of Taxol, Cremophor EL (CrEL). Besides Taxol, CrEL is widely used in the pharmaceutical industry to formulate a variety of marketed drugs such as anesthetics Propofol and immunosuppressant Cyclosporin A. Because paclitaxel is insoluble in water, CrEL played a vital role for the successful development of Taxol, the first paclitaxel-based antineoplastics (Ezrahi et al., 2019). Unfortunately, CrEL is known to induce hypersensitivity in humans, so strong that premedication with immunosuppressants such as dexamethasone is required. Nevertheless, the biological basis of CrEL-induced hypersensitivity, and other CrEL-related side effects were poorly characterized (Mielke et al., 2006; Gelderblom et al., 2001).

In the present study, we demonstrate that CrEL at clinically relevant concentrations greatly stimulates aerobic glycolysis and ANGPTL4 expression. These findings may have several implications in cancer management. We find that patients with breast cancer treated with Taxol exhibit higher levels of TGs, compared to those treated with other taxane-based chemotherapy drugs. This phenomenon may be explained by our results showing that CrEL strongly induces the expression of ANGPTL4, which is further enhanced by premedication with dexamethasone. ANGPTL4 is a major determinant of blood TG levels in humans (Aryal et al., 2019). Large-scale human genetic studies demonstrate that individuals bearing loss-of-function ANGPTL4 variants have significant lower level of TGs and reduced risk of cardiovascular diseases (Gusarova et al., 2018, Myocardial Infarction et al., 2016; Dewey et al., 2016). Conversely, enhanced ANGPTL4 expression in various mouse models consistently causes increased blood TGs and facilitates the development of metabolic disorders (Aryal et al., 2019). In our breast cancer patient cohort, the amplitude of rising TG levels correlates with the cumulative dosage of Taxol, indicating a lasting effect of CrEL on patient lipid profiles. This is consistent with early studies showing that a significant amount of CrEL persists in human plasma even at a week after Taxol infusion (Sparreboom et al., 1998). Secondly, enhanced glycolysis and ANGPTL4 expression may interfere with the efficacy of other anti-cancer drugs. Platinum-based drugs are frequently used in combination with Taxol as first-line treatment for such as lung and ovarian cancers. In our preliminary study, we found that pretreatment with CrEL confers resistance to cisplatin in cancer cells. This effect of CrEL depends on glycolysis because 2-DG restored cisplatin sensitivity, whereas oligomycin further enhanced CrEL-induced resistance (Figure S5). Similarly, CrEL also enhanced aerobic glycolysis and increased the expression of ANGPTL4 in breast cancer cell lines MDA-MB-231 and MCF7 (Figure S6). We suspect that ANGPTL4 might also contribute because it is known to promote survival pathways in cancer cells (Zhu et al., 2011).

We show that CrEL can trigger ER stress and unfolded protein response signaling (UPR). UPR is well known to stimulate proinflammatory NF-kB and JNK-AP1 pathways (Hotamisligil, 2010). This explains why dexamethasone is so effective to counteract CrEL-induced hypersensitivity in patients because dexamethasone-activated glucocorticoid receptor is a powerful suppressor of NF-kB and AP1 transcriptional activities (Mcmaster and Ray, 2008). Nevertheless, even with premedication, minor and life-threatening hypersensitivity still occurs in about 40% and 3% of patients, respectively (Hennenfent and Govindan, 2006). These uncontrolled side effects may be explained by our findings that CrEL at clinically relevant concentration can induce pyroptosis, a lytic proinflammatory form of cell death (Broz et al., 2020). The connection between CrEL and UPR may also explain Taxol-induced peripheral neuropathy (Inceoglu et al., 2015), a common side effect leading to suboptimal treatment (Hershman et al., 2013, 2018). Dexamethasone counteracts most CrEL-induced proinflammatory gene expression. However, it does not resolve UPR. Aberrant activation of UPR is known to suppress CD8^+^ T cell effector function (Franco et al., 2020; Song et al., 2018). The fact that CrEL strongly stimulates UPR may help to explain the puzzling results from recent large-scale clinical trials to treat triple-negative breast cancer (Franzoi and de Azambuja, 2020). IMpassion130 Trial using Abraxane (nanoparticle albumin-bound paclitaxel) and Atezolizumab (anti-PD-L1) was successful and instrumental for the FDA approval of this combinatory therapy (Schmid et al., 2018). Surprisingly, the most recent IMpassion131 Trial using Atezolizumab and solvent-formulated paclitaxel failed to achieve any clinical efficacy (Franzoi and de Azambuja, 2020).

### Limitations of the study

Taxol is among the most affordable and best-selling anti-cancer drugs ever produced. CrEL is the first excipient that renders paclitaxel sufficient solubility to achieve therapeutical benefit in patients. The present study uncovers the complex role of CrEL on metabolism and inflammation in cultured human cancer and primary immune cells and highlights the need to thoroughly investigate the biological effects of excipients, an overlooked gap between basic and clinical research (Pottel et al., 2020). Further investigations using animal models, such as various conditional ANGPTL4 knockout mice (Aryal et al., 2019), are needed to clarify the *in vivo* relevance of these molecular mechanisms.

## STAR★Methods

### Key resources table

**Table:** 

REAGENT or RESOURCE	SOURCE	IDENTIFIER
**Antibodies**		

HIF1α	Cell Signaling Technology	Cat# 36169
HIF1β	Cell Signaling Technology	Cat# 5537
p-mTOR(Ser2448)	Cell Signaling Technology	Cat# 5536
mTOR	Cell Signaling Technology	Cat# 2983
p-p70S6(Thr389)	Cell Signaling Technology	Cat# 9234
p-Histone H3(Ser10)	Cell Signaling Technology	Cat# 53348
p-IκBα(Ser32)	Cell Signaling Technology	Cat# 2859
IκBα	Cell Signaling Technology	Cat# 4814
p-eIF2α(Ser51)	Cell Signaling Technology	Cat# 3398
Caspase-3	Cell Signaling Technology	Cat# 9662
PARP1	Cell Signaling Technology	Cat# 9542
ANGPTL4	Abcam	Cat# ab206420
p-IRE1(Ser724)	Abcam	Cat# ab243665
GSDME	Abcam	Cat# ab215191
GSDMD	Santa Cruz	Cat# sc-81868
H3.1	Abmart	Cat# P30266M
GAPDH	KangCheng	Cat# KC-5G5
TUBULIN	Zen Bioscience	Cat# 200608

**Chemicals, peptides, and recombinant proteins**		

Cremophor EL	MCE	Cat# HY-Y1890
Tween80	MCE	Cat# HY-Y1891
2-DG	MCE	Cat# HY-13966
Oxamate	Santa Cruz	Cat# sc-215880
UK-5099	MCE	Cat# HY-15475
Oligomycin	MCE	Cat# HY-N6782
LY294002	MCE	Cat# HY-10108
INK-128 (Sapanisertib)	MCE	Cat# HY-13328
Nigericin	MCE	Cat# HY-100381
CY-09	MCE	Cat# HY-103666
VX-765	Selleck	Cat# S2228
Nec-1	Selleck	Cat# S8037
GSK-872	MCE	Cat# HY-101872
Z-VAD-FMK	Selleck	Cat# S7023
Z-DEVD-FMK	Selleck	Cat# S7312
Z-YVAD-FMK	Apexbio	Cat# A8955
Z-LEVD-FMK	Abcam	Cat# ab120489
Dexamethasone	MCE	Cat# HY-14648
Mifepristone (RU486)	Cayman	Cat# 10006317
GSK3787	MCE	Cat# HY-15577
GW9662	MCE	Cat# HY-16578
BAY85-3934 (Molidustat)	MCE	Cat# HY-12654
desferrioxamine	MCE	Cat# HY-B0988

**Critical commercial assays**		

cDNA Reverse Transcription Kit	Invitrogen	Cat# 28025021
SYBR green Mastermix	Biorad	Cat# 1725271
2-NBDG	GLPBIO	Cat# GC10289
Cell Counting Kit-8	Beyotime	Cat# C0037
FITC Annexin V Apoptosis Detection Kit	BD Biosciences	Cat# 556547
LDH Cytotoxicity Assay Kit	Beyotime	Cat# C0017
Clarity^TM^ Western ECL Substrate	Biorad	Cat# 1705062
Seahorse XF Glycolysis Stress Test Kit	Agilent	Cat# 103020-100
BCA Protein Assay Kit	ThermoFisher	Cat# 23225
Lactate Colorimetric Assay Kit	Elabscience	Cat# E-BC-K044
Human ANGPTL4 ELISA Kit	Elabscience	Cat# E-EL-H0337

**Experimental models: Cell lines**		

HEK293T	ATCC	Cat# CRL-3216
Caov-3	ATCC	Cat# HTB-75
A549	ATCC	Cat# CCL-185
HCT116	ATCC	Cat# CCL-247
MDA-MB-231	ATCC	Cat# HTB-26
MCF7	ATCC	Cat# HTB-22
HeLa	ATCC	Cat# CRM-CCL-2
RKO	ATCC	Cat# CRL-2577
THP-1	ATCC	Cat# TIB-202
THP-1 GSDMD knockout cell line	This paper	N/A
THP-1 GSDME knockout cell line	This paper	N/A
THP-1 Caspase-3 knockout cell line	This paper	N/A

**Oligonucleotides**		

RT-PCR primers (Table S1)	This paper	N/A
GSDMD-sg1 5’- CGGCCTTTGAGCGGGTAGTC -3’	This paper	N/A
GSDMD-sg2 5’- GGACCACTCTCCGGACTACC -3’	This paper	N/A
GSDME-sg1 5’- TAAGTTACAGCTTCTAAGTC -3’	This paper	N/A
GSDME-sg2 5’- AGGGTGAGGGATAAAAACTG -3’	This paper	N/A
Caspase-3-sg1 5’- GGAAGCGAATCAATGGACTC -3’	This paper	N/A
Caspase-3-sg2 5’- GTCGATGCAGCAAACCTCAGG -3’	This paper	N/A

**Recombinant DNA**		

LentiCRISPR-V2	Addgene	Cat# 52961

**Deposited data**		

RNA-seq data	This paper	PRJNA759390

**Software and algorithms**		

GraphPad Prism 8	GraphPad	N/A
ImageJ	ImageJ	N/A
FlowJo 10	FlowJo	N/A
Seahorse Wave Desktop	Agilent	N/A
FastQC	v0.11.8	N/A
Trimmomatic	v0.39	N/A
Hisat2	v2.0.5	N/A
DESeq2	v1.16.1	N/A

**Other**		

Fetal Bovine Serum	GEMINI	Cat# 900-108
Sterile PBS	HYCLONE	Cat# SH30256.01
Puromycin	Invitrogen	Cat# A1113803
RPMI 1640	Invitrogen	Cat# 11875119
DMEM	Invitrogen	Cat# 11965092
Penicillin-Streptomycin	Invitrogen	Cat# 15140122
TRIzol™ Reagent	Invitrogen	Cat# 15596026

### Resource availability

#### Lead contact

Further information and requests for resources and reagents should be directed to and will be fulfilled by the lead contact, Qintong Li (liqintong@scu.edu.cn).

#### Materials availability

All unique reagents generated in this study are available from the lead contact with a completed Material Transfer Agreement.

### Experimental model and subject details

#### Cell lines

HEK293T, Caov-3, A549, HCT116, MDA-MB-231, MCF7, HeLa, RKO and THP-1 cells were purchased from ATCC, and verified to be free of mycoplasma by the PCR method. HEK293T, Caov-3, A549, HCT116, MDA-MB-231, MCF7, HeLa and RKO cells were grown in Dulbecco’s modified Eagle’s medium (DMEM) with 10% FBS and penicillin-streptomycin. THP-1 cells were grown in RPMI-1640 medium with 10% FBS and penicillin-streptomycin. To generate macrophage-like cells, THP-1 cells were seeded at 1×10^6^ cells per well in six-well plates, and treated with 50 ng/ml phorbol myristate acetate (PMA) for 3 days. All cells were maintained at 37°C and 5% CO_2_. Most culture media were from Gibco unless otherwise noted. Heat-inactivated FBS was purchased from Gemini Bio-Products.

#### Primary cell cultures

Peripheral blood mononuclear cells (PBMCs) were enriched by leukapheresis from healthy human donors, and further purified by anti-CD14 Microbeads Human (Miltenyi Biotec) for positive selection to separate CD14+ from CD14-cells using the Automacs separator. The purities of each cell type were determined by FACS analysis using PI (BD, 51-66211E), FITC-CD45 (BD, 555482) and APC-CD14 (BD, 555399). More than 10,000 events were collected by BD FACSCelesta, and analyzed by FlowJo V10 software.

### Method details

#### Quantification of extracellular lactate level

The amount of lactate was determined using the Lactate Colorimetric Assay Kit (Elabscience), following the manufacturer’s instruction. All measurements were performed in triplicate, quantitated using analytical-grade lactate standard, and normalized to blank medium.

#### Seahorse metabolic analysis

The extracellular acidification rate (ECAR) and oxygen consumption rate (OCR) were measured by a Seahorse XFe24 Extracellular Flux Analyzer. Caov-3 cells were seeded in a Seahorse 24-well plate in DMEM supplemented with 10%FBS and treated with Cremophor EL or indicated small-molecule inhibitors. For ECAR, cells were incubated free of glucose and CO_2_ for 30 minutes as basal condition, and measured after injection of glucose (10 mM), oligomycin (1 μM) and 2-DG (50 mM), respectively. OCR were measured under basal condition and after injection of oligomycin (1 μM), FCCP (1.5 μM) and antimycin A (0.5 μM) plus rotenone (0.5 μM).

#### RNA sequencing and data analysis

Sequencing libraries were generated using NEBNext® UltraTM RNA Library Prep Kit for Illumina® (NEB, USA) following manufacturer’s recommendations, and index codes were added to attribute sequences to each sample. The library preparations were sequenced on an Illumina Novaseq platform and 150 bp paired-end reads were generated. The resulting fastq file was quality controlled using FastQC v0.11.8, and adapters were trimmed using Trimmomatic v0.39. Paired-end clean reads were aligned to the reference genome (GRCh38.p12) using Hisat2 v2.0.5. featureCounts v1.5.0-p3 was used to count the reads numbers mapped to each gene. FPKM of each gene was calculated based on the length of the gene and reads count mapped to this gene. Downstream analysis for differential gene expression of the raw count table was performed using the DESeq2 R package (1.16.1). The volcano plots of each group comparison were generated by R. Gene Set Enrichment Analysis (GSEA) was performed using the lists of hallmark gene sets from the Molecular Signature Database (MsigDB) using default parameters.

#### Immunoblotting and antibodies

Total cell lysates were prepared using lysis buffer (10 mM PIPES, pH 6.8, 100 mM NaCl, 3 mM MgCl_2_, 300 mM sucrose, 1 mM EGTA, 0.5% Triton X-100, 1 mM dithiothreitol and 1.2 mM PMSF) supplemented with protease inhibitors (Roche). Samples were briefly sonicated to break down DNA, and total protein amount was quantified by BCA protein assay (Thermo). The following antibodies were used, anti-HIF1α (Cell Signaling Technology, 36169), HIF1β (5537), AKT (9272), p-mTOR (5536), mTOR (2983), p-p70S6 (9234), p-Histone H3 (53348), p-IκBα (2859), IκBα (4814), p-eIF2α (3398), Caspase-3 (9662) and PARP1 (9542); anti-ANGPTL4 (Abcam, ab206420), p-IRE1 (ab243665), GSDME (ab215191); anti-GSDMD (Santa Cruz, sc-81868); anti-p-AKT (Abways, P31749); anti-H3.1 (Abmart, P30266M); anti-GAPDH (KangCheng, KC-5G5) and anti-TUBULIN (Zen Bioscience, 200608). Quantification of the bands was performed using ImageJ software.

#### Glucose uptake assay

THP-1 or Caov-3 cells were first treated with indicated concentration of Cremophor EL overnight. Cells were washed in DMEM without glucose, followed by incubation with glucose-free medium for 2 hours. Culture medium was then removed from each well, and treated with medium with or without 200 μM 2-deoxy-2-[(7-nitro-2,1,3- benzoxadiazol-4-yl)amino]-D-glucose (2-NBDG; GlpBio) for 20 minutes. Samples were washed once in PBS, and analyzed by BD FACSCelesta flow cytometer for at least 10,000 events per sample.

#### qPCR analysis

Total RNA was isolated using the TRIzol™ Reagent (Invitrogen), reverse transcribed using oligo dT and M-MLV Reverse Transcriptse (Invitrogen), and cDNA was amplified using SYBR green Mastermix (Biorad) on Biorad CFX96. The data were presented as the fold change in gene expression normalized to HPRT1. PCR primers were designed using online IDT primer tools. Primer sequences are provided in Table S1.

#### Clinical data analysis

The electronic medical records of breast cancer patients between 2009 and 2018 were retrospectively collected from the Department of Breast Surgery, West China Hospital, Sichuan University. This retrospective study was approved by the Institutional Review Board and Ethics Committee of West China Hospital, Sichuan University, and informed consent was obtained from all patients. Whole blood lipid profiles pre- and post-chemotherapy were determined by College of American Pathologists (CAP)-accredited laboratory in West China Hospital (Sichuan University) using Cobas8000 system (Roche Diagnostics GmbH, Germany). For pre-chemotherapy, whole-blood samples were collected within a week before the first cycle of chemotherapy. For post-chemotherapy, samples were collected within 2 weeks after the end of the last cycle of chemotherapy. Based on guidelines for the management of dyslipidemia patients in China (33), the following cut-off values were set as upper limit of normal level for triglycerides (TG, 1.7 mM), total cholesterols (TC, 5.2 mM), and low-density lipoprotein (LDL-C, 3.4 mM), and lower limit of normal level for high density lipoprotein (HDL-C, 1 mM).

#### Cytotoxicity assay and cytokine measurement

Cell viability was determined by the Cell Counting Kit-8 (Beyotime), and cytotoxicity was measured by the LDH assay. LDH in culture medium was measured using the LDH Cytotoxicity Assay Kit (Beyotime). Cytotoxicity was defined as the percentage of released LDH compared with maximal LDH activity after cell lysis with 1% Triton X-100. For FACS analysis, cells were stained using FITC Annexin V Apoptosis Detection Kit I (BD Biosciences), and the data were analyzed using FlowJo V10 software. ANGPTL4 and IL-1β proteins were quantified in culture medium by ELISA.

#### Constructs and generation of knockout cell lines

CRISPR-mediated knockout plasmids containing guide RNAs were generated in LentiCRISPR-V2 (Addgene, #52961) according to the standard protocol. gRNA sequences for GSDMD, GSDME and Caspase-3 were listed in Table S1. Two gRNAs were simultaneously transduced in cells to target indicated genes, and colonies were screened by protein blotting analysis to identify GSDMD, GSDME or Caspase-3 knockout cells.

### Quantification and statistical analysis

For western blots, representative images were shown with at least two independent experiments. ECAR and OCR experiments are shown as single experiment with biologically independent replicates (n = 3). Real-time qPCR and all other experiments were repeated multiple times and results are shown as mean ± s.d. values (n > 3). Data, excluding those describing transcriptomics or metabolomics data, were analyzed and presented with GraphPad Prism 8 software. Data were log-transformed in case data were not normally distributed. Statistical analyses were performed by unpaired Student's t-test unless otherwise specified. The statistical significance of difference was set at P < 0.05.

## Data Availability

• RNA-seq data have been deposited at SRA database under accession number: PRJNA759390. All data reported in this paper will be shared by the lead contact upon request. • This study did not generate original code. • Any additional information required to reanalyze the data reported in this paper is available from the lead contact upon request.

## References

[bib1] Adams J.D., Flora K.P., Goldspiel B.R., Wilson J.W., Arbuck S.G., Finley R. (1993). Taxol: a history of pharmaceutical development and current pharmaceutical concerns. J. Natl. Cancer Inst. Monogr..

[bib2] Aryal B., Price N.L., Suarez Y., Fernandez-Hernando C. (2019). ANGPTL4 in metabolic and cardiovascular disease. Trends Mol. Med..

[bib3] Broz P., Pelegrin P., Shao F. (2020). The gasdermins, a protein family executing cell death and inflammation. Nat. Rev. Immunol..

[bib4] Cain D.W., Cidlowski J.A. (2017). Immune regulation by glucocorticoids. Nat. Rev. Immunol..

[bib5] Copp J., Manning G., Hunter T. (2009). TORC-specific phosphorylation of mammalian target of rapamycin (mTOR): phospho-Ser2481 is a marker for intact mTOR signaling complex 2. Cancer Res..

[bib6] Dewey F.E., Gusarova V., O'Dushlaine C., Gottesman O., Trejos J., Hunt C., van Hout C.V., Habegger L., Buckler D., Lai K.M. (2016). Inactivating variants in ANGPTL4 and risk of coronary artery disease. N. Engl. J. Med..

[bib7] Divakaruni A.S., Paradyse A., Ferrick D.A., Murphy A.N., Jastroch M. (2014). Analysis and interpretation of microplate-based oxygen consumption and pH data. Methods Enzymol..

[bib8] Ezrahi S., Aserin A., Garti N. (2019). Basic principles of drug delivery systems - the case of paclitaxel. Adv. Colloid Interfaces Sci..

[bib9] Fingar D.C., Inoki K. (2012). Deconvolution of mTORC2 "in silico. Sci. Signal..

[bib10] Franco F., Jaccard A., Romero P., Yu Y.R., Ho P.C. (2020). Metabolic and epigenetic regulation of T-cell exhaustion. Nat. Metab..

[bib11] Franzoi M.A., de Azambuja E. (2020). Atezolizumab in metastatic triple-negative breast cancer: IMpassion130 and 131 trials - how to explain different results?. ESMO Open.

[bib12] Gelderblom H., Verweij J., Nooter K., Sparreboom A. (2001). Cremophor EL: the drawbacks and advantages of vehicle selection for drug formulation. Eur. J. Cancer.

[bib13] Gradishar W.J., Tjulandin S., Davidson N., Shaw H., Desai N., Bhar P., Hawkins M., O'Shaughnessy J. (2005). Phase III trial of nanoparticle albumin-bound paclitaxel compared with polyethylated castor oil-based paclitaxel in women with breast cancer. J. Clin. Oncol..

[bib14] Gusarova V., O'Dushlaine C., Teslovich T.M., Benotti P.N., Mirshahi T., Gottesman O., van Hout C.V., Murray M.F., Mahajan A., Nielsen J.B. (2018). Genetic inactivation of ANGPTL4 improves glucose homeostasis and is associated with reduced risk of diabetes. Nat. Commun..

[bib15] Haschka M., Karbon G., Fava L.L., Villunger A. (2018). Perturbing mitosis for anti-cancer therapy: is cell death the only answer?. EMBO Rep..

[bib16] Hennenfent K.L., Govindan R. (2006). Novel formulations of taxanes: a review. Old wine in a new bottle?. Ann. Oncol..

[bib17] Hershman D.L., Unger J.M., Crew K.D., Minasian L.M., Awad D., Moinpour C.M., Hansen L., Lew D.L., Greenlee H., Fehrenbacher L. (2013). Randomized double-blind placebo-controlled trial of acetyl-L-carnitine for the prevention of taxane-induced neuropathy in women undergoing adjuvant breast cancer therapy. J. Clin. Oncol..

[bib18] Hershman D.L., Unger J.M., Crew K.D., Till C., Greenlee H., Minasian L.M., Moinpour C.M., Lew D.L., Fehrenbacher L., Wade J.L. (2018). Two-year trends of taxane-induced neuropathy in women enrolled in a randomized trial of acetyl-L-carnitine (SWOG S0715). J. Natl. Cancer Inst..

[bib19] Hetz C., Zhang K., kaufman R.J. (2020). Mechanisms, regulation and functions of the unfolded protein response. Nat. Rev. Mol. Cell Biol..

[bib20] Hotamisligil G.S. (2010). Endoplasmic reticulum stress and the inflammatory basis of metabolic disease. Cell.

[bib21] Ilinskaya A.N., Clogston J.D., Mcneil S.E., Dobrovolskaia M.A. (2015). Induction of oxidative stress by Taxol(R) vehicle Cremophor-EL triggers production of interleukin-8 by peripheral blood mononuclear cells through the mechanism not requiring de novo synthesis of mRNA. Nanomedicine.

[bib22] Inceoglu B., Bettaieb A., Trindade da Silva C.A., Lee K.S., Haj F.G., Hammock B.D. (2015). Endoplasmic reticulum stress in the peripheral nervous system is a significant driver of neuropathic pain. Proc. Natl. Acad. Sci. U S A.

[bib23] Joint Committee for Guideline R. (2018). 2016 Chinese guidelines for the management of dyslipidemia in adults. J. Geriatr. Cardiol..

[bib24] Jordan M.A., Toso R.J., Thrower D., Wilson L. (1993). Mechanism of mitotic block and inhibition of cell proliferation by taxol at low concentrations. Proc. Natl. Acad. Sci. U S A.

[bib25] Karin M. (1999). How NF-kappaB is activated: the role of the IkappaB kinase (IKK) complex. Oncogene.

[bib26] Kavallaris M. (2010). Microtubules and resistance to tubulin-binding agents. Nat. Rev. Cancer.

[bib27] Kayagaki N., Stowe I.B., Lee B.L., O'Rourke K., Anderson K., Warming S., Cuellar T., Haley B., Roose-Girma M., Phung Q.T. (2015). Caspase-11 cleaves gasdermin D for non-canonical inflammasome signalling. Nature.

[bib28] Kersten S., Mandard S., Tan N.S., Escher P., Metzger D., Chambon P., Gonzalez F.J., Desvergne B., Wahli W. (2000). Characterization of the fasting-induced adipose factor FIAF, a novel peroxisome proliferator-activated receptor target gene. J. Biol. Chem..

[bib29] Liberzon A., Birger C., Thorvaldsdottir H., Ghandi M., Mesirov J.P., Tamayo P. (2015). The Molecular Signatures Database (MSigDB) hallmark gene set collection. Cell Syst..

[bib30] Mcmaster A., Ray D.W. (2008). Drug insight: selective agonists and antagonists of the glucocorticoid receptor. Nat. Clin. Pract. Endocrinol. Metab..

[bib31] Mielke S., Sparreboom A., Mross K. (2006). Peripheral neuropathy: a persisting challenge in paclitaxel-based regimes. Eur. J. Cancer.

[bib32] Mossmann D., Park S., Hall M.N. (2018). mTOR signalling and cellular metabolism are mutual determinants in cancer. Nat. Rev. Cancer.

[bib33] Myocardial Infarction G., Investigators C.A.E.C., Stitziel N.O., Stirrups K.E., Masca N.G., Erdmann J., Ferrario P.G., Konig I.R., Weeke P.E., Webb T.R. (2016). Coding variation in ANGPTL4, LPL, and SVEP1 and the risk of coronary disease. N. Engl. J. Med..

[bib34] Poruchynsky M.S., Komlodi-Pasztor E., Trostel S., Wilkerson J., Regairaz M., Pommier Y., Zhang X., Kumar MAITY T., Robey R., Burotto M. (2015). Microtubule-targeting agents augment the toxicity of DNA-damaging agents by disrupting intracellular trafficking of DNA repair proteins. Proc. Natl. Acad. Sci. U S A.

[bib35] Pottel J., Armstrong D., Zou L., Fekete A., Huang X.P., Torosyan H., Bednarczyk D., Whitebread S., Bhhatarai B., Liang G. (2020). The activities of drug inactive ingredients on biological targets. Science.

[bib36] Rischin D., Webster L.K., Millward M.J., Linahan B.M., Toner G.C., Woollett A.M., Morton C.G., Bishop J.F. (1996). Cremophor pharmacokinetics in patients receiving 3-, 6-, and 24-hour infusions of paclitaxel. J. Natl. Cancer Inst..

[bib37] Rogers C., Fernandes-Alnemri T., Mayes L., Alnemri D., Cingolani G., Alnemri E.S. (2017). Cleavage of DFNA5 by caspase-3 during apoptosis mediates progression to secondary necrotic/pyroptotic cell death. Nat. Commun..

[bib38] Schmid P., Adams S., Rugo H.S., Schneeweiss A., Barrios C.H., Iwata H., Dieras V., Hegg R., Im S.A., Shaw Wright G. (2018). Atezolizumab and Nab-paclitaxel in advanced triple-negative breast cancer. N. Engl. J. Med..

[bib39] Semenza G.L. (2012). Hypoxia-inducible factors in physiology and medicine. Cell.

[bib40] Shi J., Zhao Y., Wang K., Shi X., Wang Y., Huang H., Zhuang Y., Cai T., Wang F., Shao F. (2015). Cleavage of GSDMD by inflammatory caspases determines pyroptotic cell death. Nature.

[bib41] Song M., Sandoval T.A., Chae C.S., Chopra S., Tan C., Rutkowski M.R., Raundhal M., Chaurio R.A., Payne K.K., Konrad C. (2018). IRE1alpha-XBP1 controls T cell function in ovarian cancer by regulating mitochondrial activity. Nature.

[bib42] Sparreboom A., van Zuylen L., Brouwer E., Loos W.J., de Bruijn P., Gelderblom H., pillay M., Nooter K., Stoter G., Verweij J. (1999). Cremophor EL-mediated alteration of paclitaxel distribution in human blood: clinical pharmacokinetic implications. Cancer Res..

[bib43] Sparreboom A., Verweij J., van der Burg M.E., Loos W.J., Brouwer E., Vigano L., Locatelli A., de Vos A.I., Nooter K., Stoter G., Gianni L. (1998). Disposition of Cremophor EL in humans limits the potential for modulation of the multidrug resistance phenotype in vivo. Clin. Cancer Res..

[bib44] Szebeni J., Muggia F.M., Alving C.R. (1998). Complement activation by Cremophor EL as a possible contributor to hypersensitivity to paclitaxel: an in vitro study. J. Natl. Cancer Inst..

[bib45] Wang Y., Gao W., Shi X., Ding J., Liu W., He H., Wang K., Shao F. (2017). Chemotherapy drugs induce pyroptosis through caspase-3 cleavage of a gasdermin. Nature.

[bib46] Weiss R.B., Donehower R.C., Wiernik P.H., Ohnuma T., Gralla R.J., Trump D.L., Baker J.R., van Echo D.A., von Hoff D.D., Leyland-Jones B. (1990). Hypersensitivity reactions from taxol. J. Clin. Oncol..

[bib47] Weiszhar Z., Czucz J., Revesz C., Rosivall L., Szebeni J., Rozsnyay Z. (2012). Complement activation by polyethoxylated pharmaceutical surfactants: Cremophor-EL, Tween-80 and Tween-20. Eur. J. Pharm. Sci..

[bib48] Yoon J.C., Chickering T.W., Rosen E.D., Dussault B., Qin Y., Soukas A., Friedman J.M., Holmes W.E., Spiegelman B.M. (2000). Peroxisome proliferator-activated receptor gamma target gene encoding a novel angiopoietin-related protein associated with adipose differentiation. Mol. Cell Biol..

[bib49] Zasadil L.M., Andersen K.A., Yeum D., Rocque G.B., Wilke L.G., Tevaarwerk A.J., Raines R.T., Burkard M.E., Weaver B.A. (2014). Cytotoxicity of paclitaxel in breast cancer is due to chromosome missegregation on multipolar spindles. Sci. Transl Med..

[bib50] Zhu P., Tan M.J., Huang R.L., Tan C.K., Chong H.C., Pal M., Lam C.R., Boukamp P., Pan J.Y., Tan S.H. (2011). Angiopoietin-like 4 protein elevates the prosurvival intracellular O_2_(-):H_2_O_2_ ratio and confers anoikis resistance to tumors. Cancer Cell.

