# Supplementary material for: Excipient of paclitaxel induces metabolic dysregulation and unfolded protein response
Source: iScience. 2021 Sep 25;24(10):103170. doi: 10.1016/j.isci.2021.103170 (PMC8501768; doi:10.1016/j.isci.2021.103170)
Supplement: Document S1. Figures S1–S7 and Table S1 [file mmc1.pdf]

iScience, Volume *24*

Supplemental information

Excipient of paclitaxel induces metabolic dysregulation and unfolded protein response

Qian Dai, Xiaolin Liu, Tao He, Chao Yang, Jinfeng Jiang, Yin Fang, Zhoukai Fu, Yuan Yuan, Shujun Bai, Tong Qiu, Rutie Yin, Ping Ding, Jie Chen, and Qintong Li

SUPPLEMENTAL INFORMATION

Figure S1. CrEL promotes glycolysis via mTOR signaling pathway, Related to Figure 1 and Figure 2

Figure S2. Retrospective analysis of patient blood lipid profile, Related to Figure 4

Figure S3. CrEL induces unfolded protein response, Related to Figure 5

Figure S4. CrEL induces pyroptosis, Related to Figure 6

Figure S5. Effect of CrEL on cisplatin-induced cell death, Related to Figure 1

Figure S6. Effect of CrEL in breast cancer cells, Related to Figure 1 and Figure 5

Figure S7. Full images of western blots, Related to Figures 1-6 and S1-6

Table S1. qPCR primers, Related to STAR Methods

Figure S1. CrEL promotes glycolysis via mTOR signaling pathway, Related to Figure 1 and Figure 2

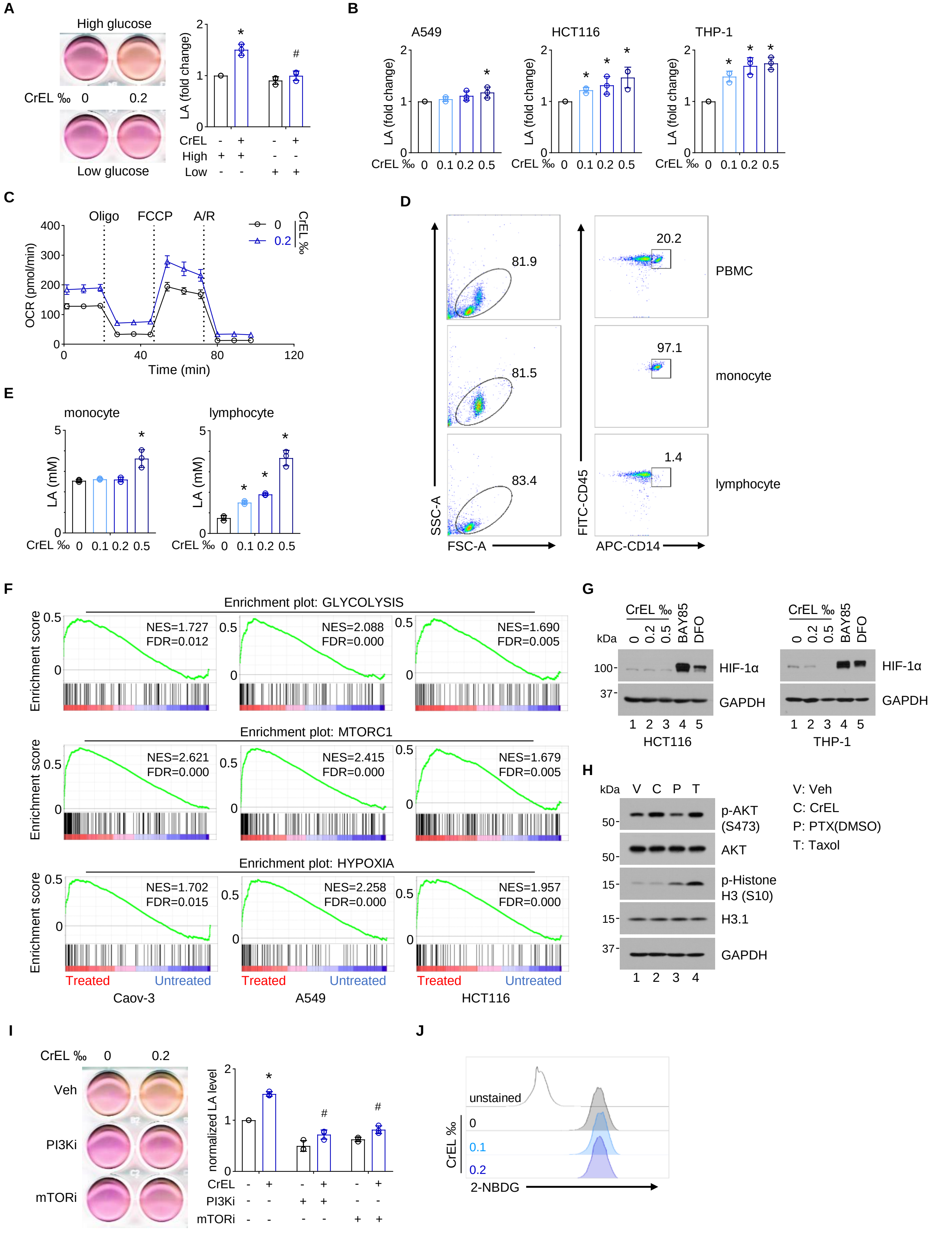

Figure S2. Retrospective analysis of patient blood lipid profile, Related to Figure 4

A

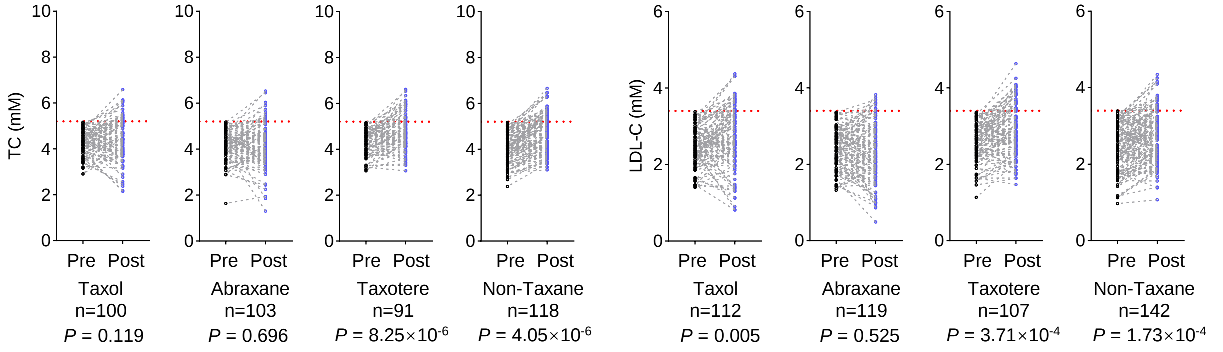

B

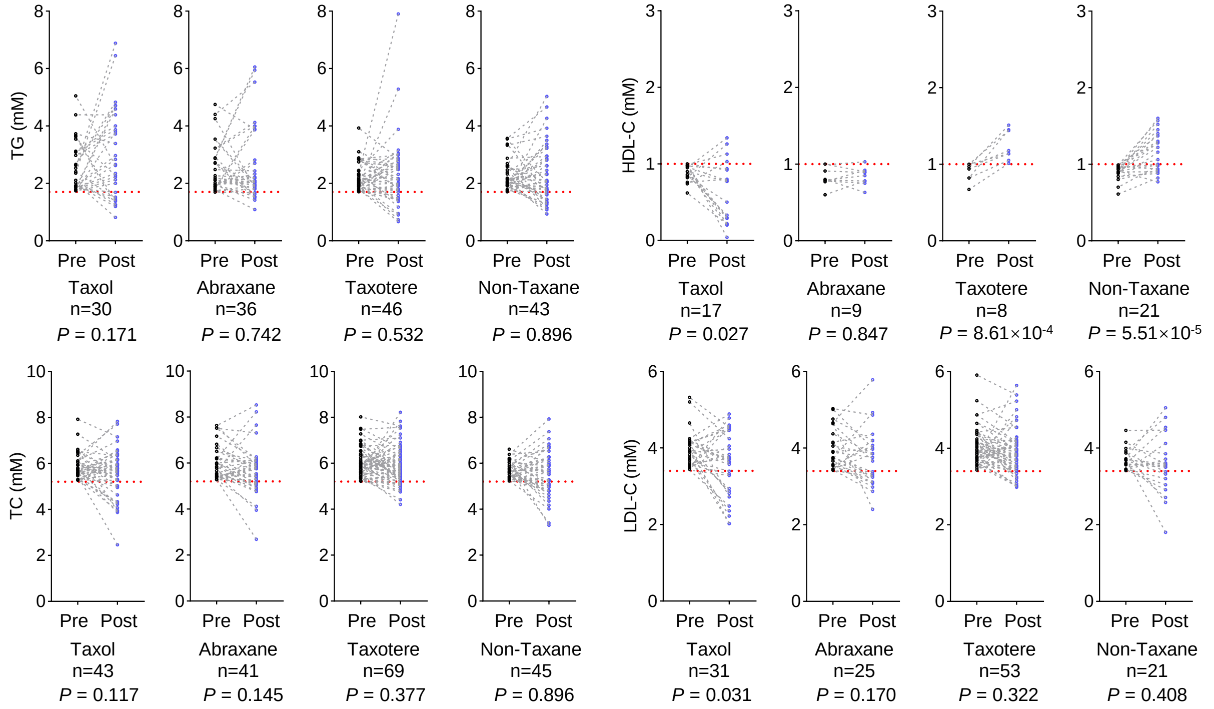

Figure S3. CrEL induces unfolded protein response, Related to Figure 5

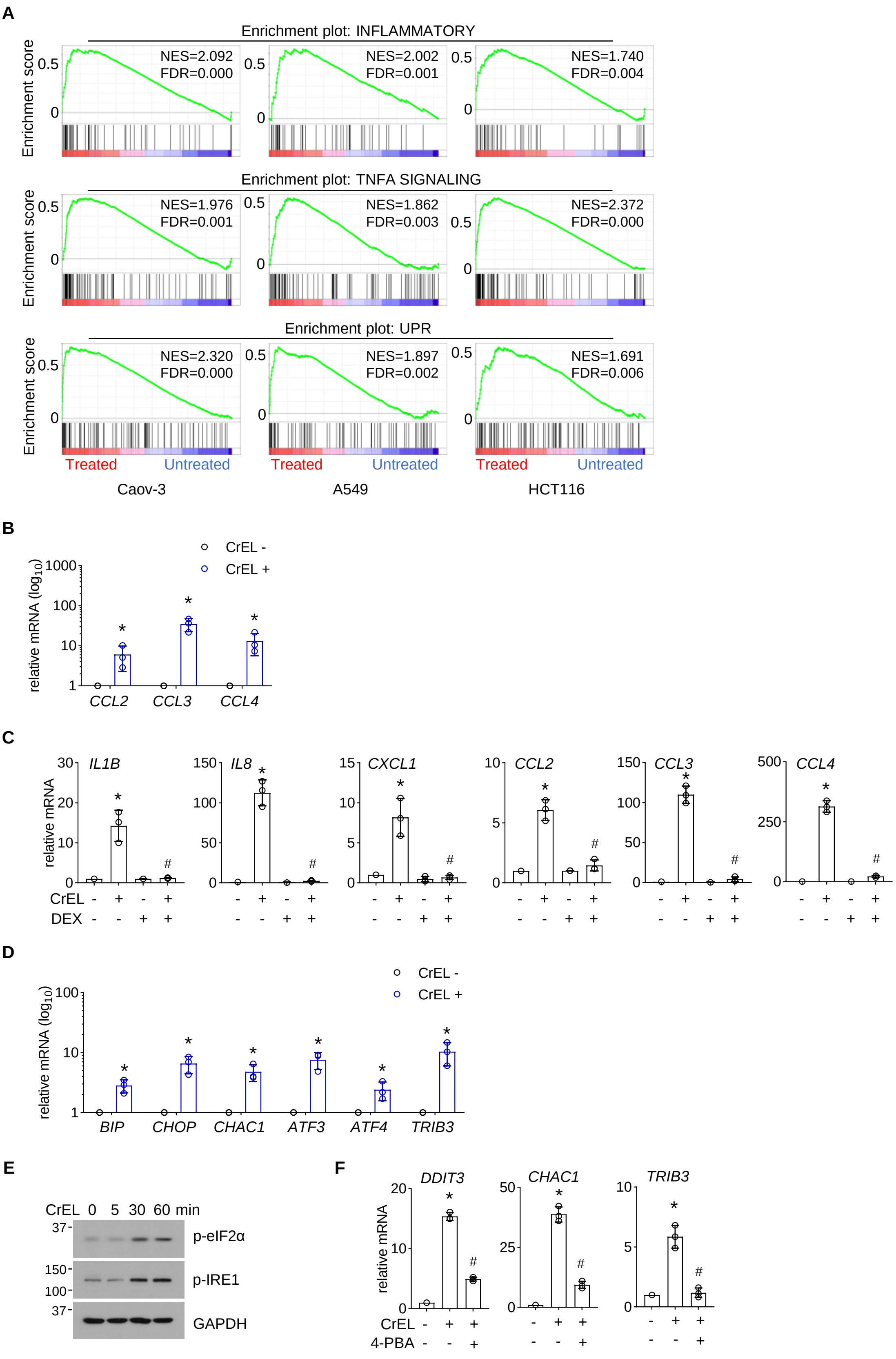

Figure S4. CrEL induces pyroptosis, Related to Figure 6

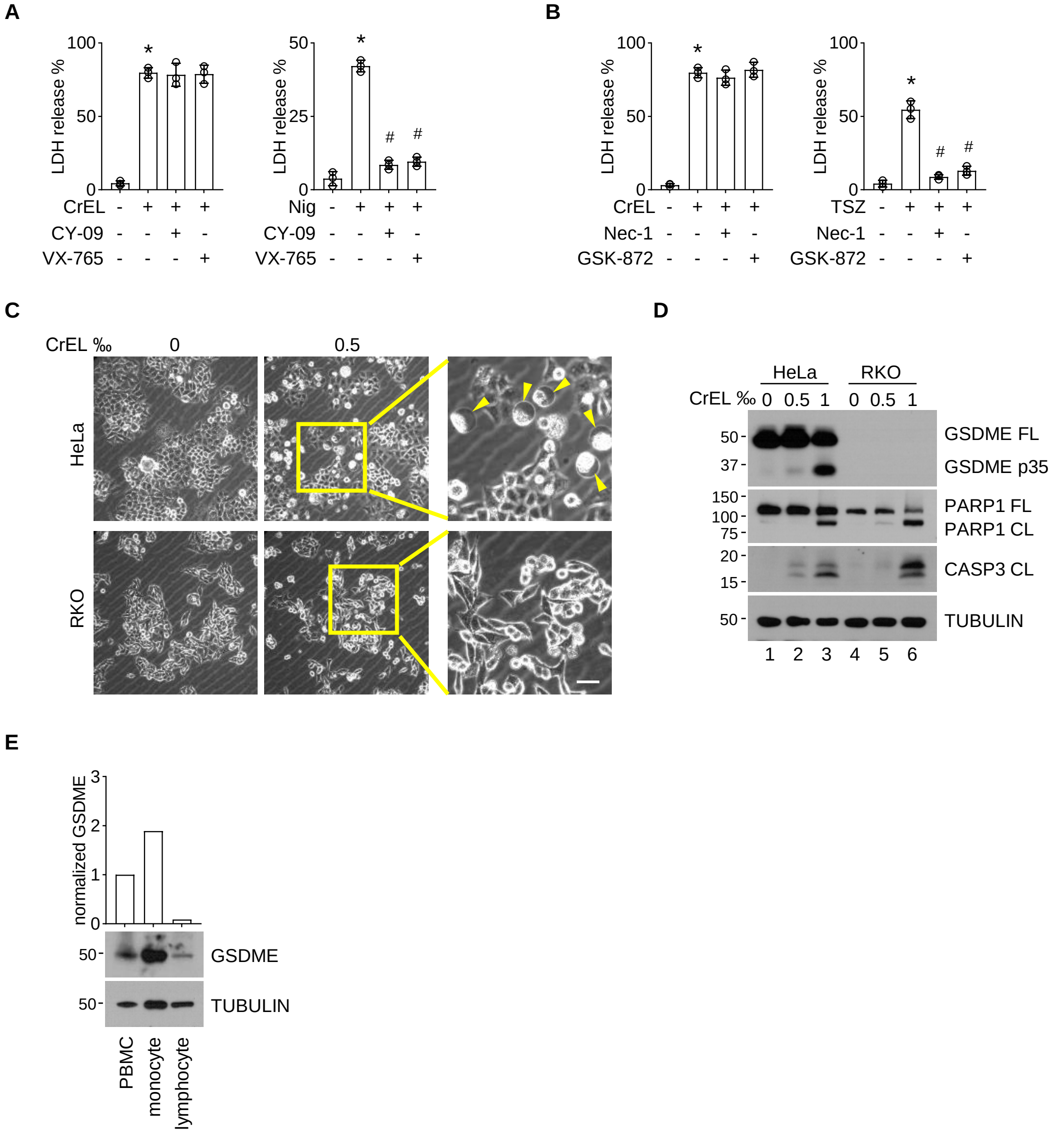

Figure S5. Effect of CrEL on cisplatin-induced cell death, Related to Figure 1

A

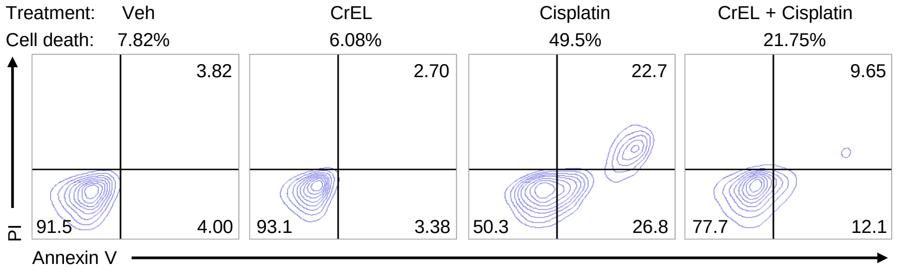

B

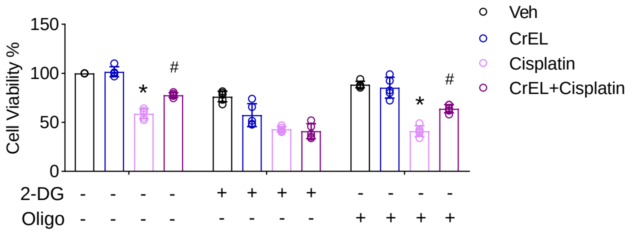

Figure S6. Effect of CrEL in breast cancer cells, Related to Figure 1 and Figure 5

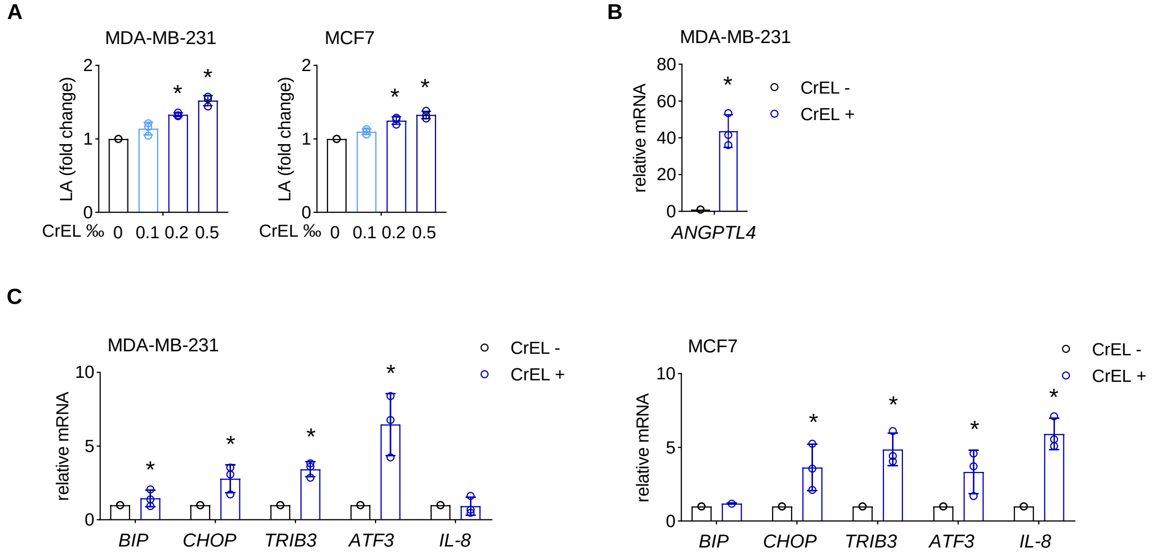

Figure S7. Full images of western blots, Related to Figures 1-6 and S1-6

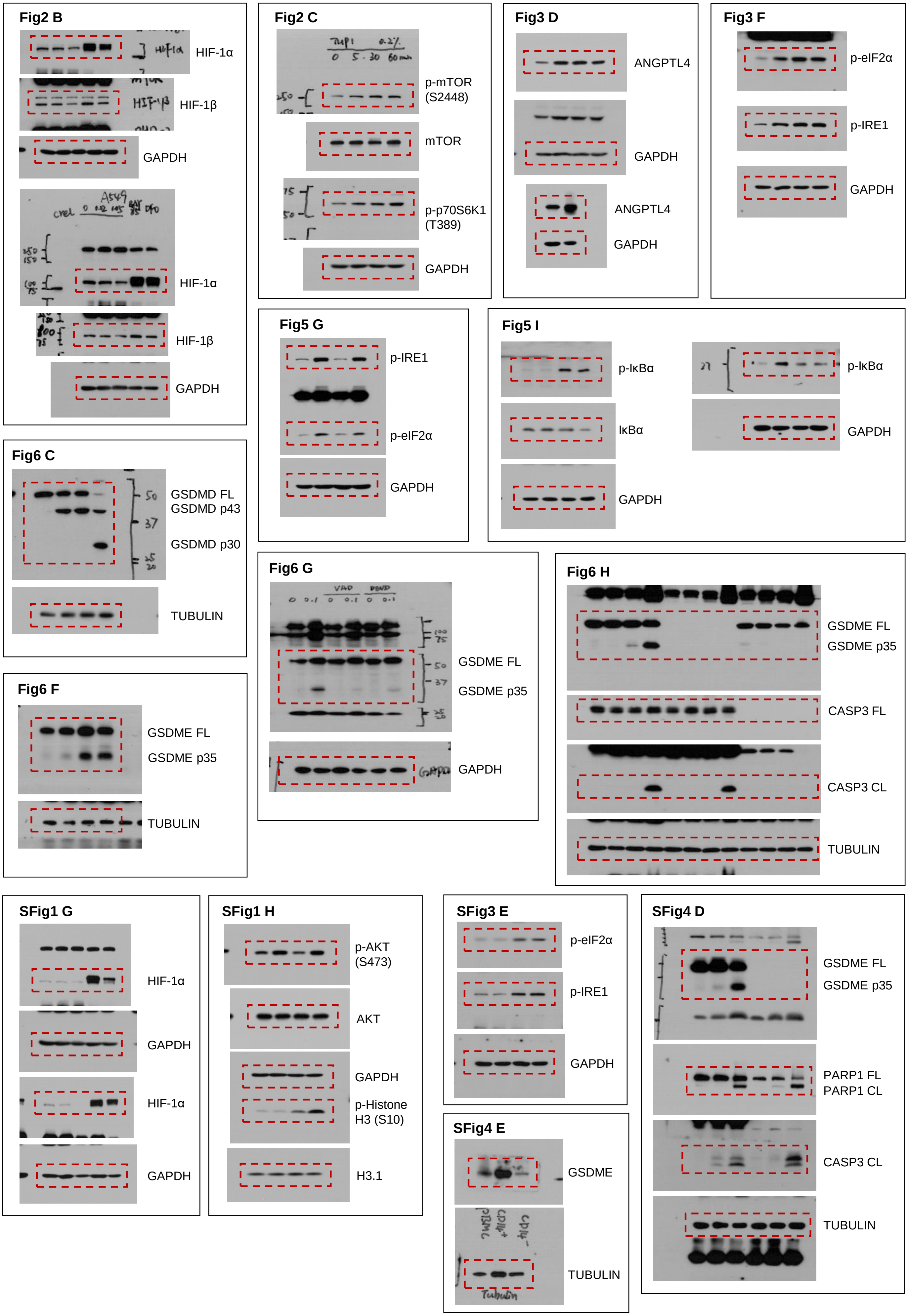

Table S1. qPCR primers, Related to STAR Methods

***HPRT1*:**<br>Forward: TGCTGAGGATTTGGAAAGGG<br>Reverse: ACAGAGGGCTACAATGTGATG
ANGPTL4:<br>Forward: AGACACAACTCAAGGCTCAG<br>Reverse: CTCATGGTCTAGGTGCTTGTG
IL1B:<br>Forward: ATGCACCTGTACGATCACTG<br>Reverse: ACAAAGGACATGGAGAACACC
IL-6:<br>Forward: CCACTCACCTCTTCAGAACG<br>Reverse: CATCTTTGGAAGGTTCAGGTTG
IL-8:<br>Forward: AGCCTTCCTGATTTCTGCAG<br>Reverse: CATCTTTGGAAGGTTCAGGTTG
IL-12A:<br>Forward: CTCCAGACCCAGGAATGTTC<br>Reverse: ATCTCTTCAGAAGTGCAAGGG
CXCL1:<br>Forward: AACCGAAGTCATAGCCACAC<br>Reverse: CCTCCCTTCTGGTCAGTTG
CCL2:<br>Forward: TGTCCCAAAGAAGCTGTGATC<br>Reverse: ATTCTTGGGTTGTGGAGTGAG
CCL3:<br>Forward: ACCAGTTCTCTGCATCACTTG<br>Reverse: TCGCTTGGTTAGGAAGATGAC
CCL4:<br>Forward: TCCTCGCAACTTTGTGGTAG<br>Reverse: TTCAGTTCCAGGTCATACACG
BIP:<br>Forward: CTGCCATGGTTCTCACTAAAATG<br>Reverse: TTAGGCCAGCAATAGTTCCAG
GRP94:<br>Forward: AAACGGGCAAGGACATCTC<br>Reverse: AAACCACAGCAAGATCCAAAAC
CHOP:<br>Forward: GTACCTATGTTTCACCTCCTGG<br>Reverse: TGGAATCTGGAGAGTGAGGG
CHAC1:<br>Forward: TTGAAGATCATGAGGGCTGC<br>Reverse: GCAAGTATTCAAGGTTGTGGC

ATF3:<br>Forward: AGAAGGAACATTGCAGAGCTAAG<br>Reverse: GGATTCTAGAGGTACACAGGAAG
ATF4:<br>Forward: CCAAGCACTTCAAACCTCATG<br>Reverse: ATCCATTTTCTCCAACATCCAATC
TRIB3:<br>Forward: TGATCTCAAGCTGTGTCGC<br>Reverse: AGTATCTCAGGTCCCACGTAG
JUN:<br>Forward: AGCCCAAACTAACCTCACG<br>Reverse: TGCTCTGTTTCAGGATCTTGG
